# Canavanine-Induced Decrease in Nitric Oxide Synthesis Alters Activity of Antioxidant System but Does Not Impact S-Nitrosoglutathione Catabolism in Tomato Roots

**DOI:** 10.3389/fpls.2019.01077

**Published:** 2019-09-20

**Authors:** Pawel Staszek, Urszula Krasuska, Katarzyna Otulak-Kozieł, Joerg Fettke, Agnieszka Gniazdowska

**Affiliations:** ^1^Department of Plant Physiology, Warsaw University of Life Sciences–SGGW, Warsaw, Poland; ^2^Department of Botany, Warsaw University of Life Sciences–SGGW, Warsaw, Poland; ^3^Biopolymer Analytics, University of Potsdam, Potsdam-Golm, Germany

**Keywords:** canavanine, cellular antioxidant system, GSNOR—GSNO reductase, nitrated proteins, nitric oxide—NO, nonproteinogenic amino acid, NOS-like activity, reactive nitrogen species (RNS)

## Abstract

Canavanine (CAN) is a nonproteinogenic amino acid synthesized in legumes. In mammalians, as arginine analogue, it is an inhibitor of nitric oxide synthase (NOS) activity. The aim of this study was to investigate the impact of CAN-induced nitric oxide level limitation on the antioxidant system and *S*-nitrosoglutathione (GSNO) metabolism in roots of tomato seedlings. Treatment with CAN (10 or 50 µM) for 24–72 h led to restriction in root growth. Arginine-dependent NOS-like activity was almost completely inhibited, demonstrating direct effect of CAN action. CAN increased total antioxidant capacity and the level of sulphydryl groups. Catalase (CAT) and superoxide dismutase (SOD) activity decreased in CAN exposed roots. CAN supplementation resulted in the decrease of transcript levels of genes coding CAT (with the exception of *CAT1*). Genes coding SOD (except *MnSOD* and *CuSOD*) were upregulated by CAN short treatment; prolonged exposition to 50-µM CAN resulted in downregulation of *FeSOD, CuSOD*, and *SODP-2*. Activity of glutathione reductase dropped down after short-term (10-µM CAN) supplementation, while glutathione peroxidase activity was not affected. Transcript levels of glutathione reductase genes declined in response to CAN. Genes coding glutathione peroxidase were upregulated by 50-µM CAN, while 10-µM CAN downregulated *GSHPx1*. Inhibition of NOS-like activity by CAN resulted in lower GSNO accumulation in root tips. Activity of GSNO reductase was decreased by short-term supplementation with CAN. In contrast, GSNO reductase protein abundance was higher, while transcript levels were slightly altered in roots exposed to CAN. This is the first report on identification of differentially nitrated proteins in response to supplementation with nonproteinogenic amino acid. Among nitrated proteins differentially modified by CAN, seed storage proteins (after short-term CAN treatment) and components of the cellular redox system (after prolonged CAN supplementation) were identified. The findings demonstrate that due to inhibition of NOS-like activity, CAN leads to modification in antioxidant system. Limitation in GSNO level is due to lower nitric oxide formation, while GSNO catabolism is less affected. We demonstrated that monodehydroascorbate reductase, activity of which is inhibited in roots of CAN-treated plants, is the protein preferentially modified by tyrosine nitration.

## Introduction

Canavanine (CAN) is a nonproteinogenic amino acid (NPAA) of a legume plant origin (Rosenthal, 1982). It is a structural analogue of L-arginine (Arg), thus its primary mode of action is linked to disturbances in Arg-dependent reactions or incorporation into proteins instead of Arg [review by Staszek et al. (2017)]. Incorporation of CAN disturbs the correct structure of target proteins and leads to protein dysfunction or prevention of repair of DNA damage. Some studies have revealed that CAN may reduce proliferation of tumor cells (Nurcahyanti and Wink, 2016). CAN is also a selective inhibitor of an inducible isoform of nitric oxide (NO) synthase (NOS), an enzyme involved in the generation of NO from Arg in mammalians (Abd el-Gawad and Khalifa, 2001; Li et al., 2001). CAN-dependent restriction of NOS activity was accompanied by a limitation in NO emission (Luzzi and Marletta, 2005), and induction of an oxidative burst (Demiryürek et al., 1997; Riganti et al., 2003).

Based on experiments conducted in our laboratory, we have suggested that also in plant research, CAN may be used as a convenient biochemical tool for the modification of NO metabolism (Krasuska et al., 2016c; Staszek et al., 2017). Arg-dependent formation of NO in plants is still under investigation and discussion, although there are many reports confirming contribution of this oxidative pathway of NO synthesis in various plant tissues (review by Jeandroz et al., 2016 and Corpas and Barroso, 2017).

Over the past two decades, due to publication of numerous reports, NO has been accepted as a signaling molecule in plants development and responses to stresses (review by Baudouin and Hancock, 2014 and Domingos et al., 2015). It is widely accepted that the activity of reactive nitrogen species (RNS), including NO, should be considered on the basis of modifications in reactive oxygen species (ROS) metabolism (Corpas and Barroso, 2013; Groß et al., 2013; Begara-Morales et al., 2016; Farnese et al., 2016).

**Figure 1:**
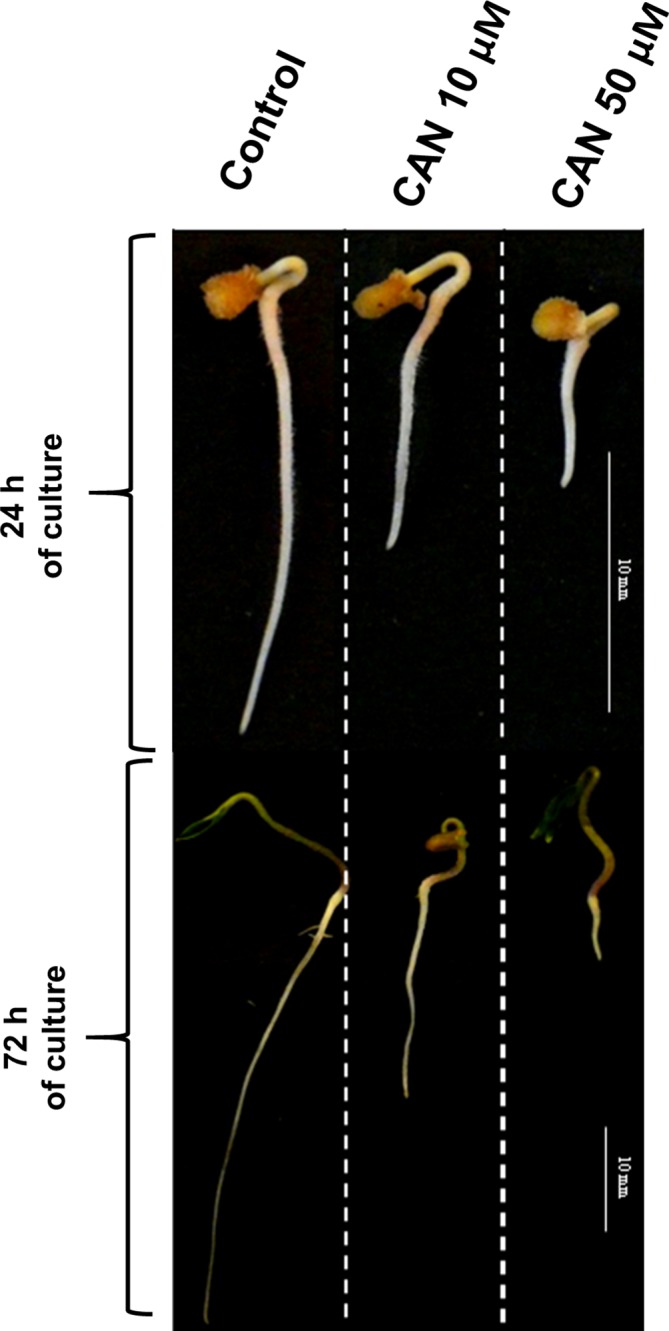
Tomato seedlings grown for 24 or 72 h in water (control) or CAN aqueous solutions (10 or 50 µM). Images of representative seedlings are presented.

In our previous experiments, tomato (*Solanum lycopersicum* L.) seedlings were cultured for 24 or 72 h in 10-µM or 50-µM CAN resulting in inhibition of root growth in 50 or 100%, respectively (**Figure 1**). Malformations in root morphology (shorter and thicker roots, limited number of lateral roots) were accompanied by modification in NO and peroxynitrite (ONOO^-^) production and localization (Krasuska et al., 2016a; Krasuska et al., 2016b). CAN restricted NO emission and transiently (after 24 h) enhanced ONOO^-^ production suggesting direct impact of this NPAA on NO biosynthesis (Krasuska et al., 2016a; Krasuska et al., 2016b). In the current work, we have measured Arg-dependent NOS-like activity in tomato roots to prove that CAN is an inhibitor of Arg-dependent NO formation in plants.

Supplementation of tomato seedlings with CAN led to increased superoxide radical (O_2_^•-^) generation and elevated hydrogen peroxide (H_2_O_2_) concentration in the roots, accompanied by stimulation of protein carbonyl groups formation (Krasuska et al., 2016a). Based on these data, we have proposed that CAN-induced oxidative burst could be potentially due to the disruption of the cellular antioxidant system, as NO regulates many enzymes involved in modulation of ROS level (Begara-Morales et al., 2016). The aims of this work were to a) analyze the impact of CAN-induced NO limitation on activity of the enzymatic antioxidant system: superoxide dismutase (SOD; EC 1.15.1.1), catalase (CAT; EC1.11.1.6), glutathione (GSH) reductase (GR; EC 1.8.1.7), and GSH peroxidase (GPx; EC 1.11.1.9) and to b) examine how CAN influences cellular antioxidants: total content of thiols and total antioxidant capacity.

The intracellular level of NO depends on its biosynthesis and detoxification (Astier et al., 2018; Lindermayr, 2018). NO and GSH form nitrosoglutathione (GSNO). The GSNO pool is regulated by the activity of GSNO reductase (GSNOR; EC 1.2.1.1), which converts GSNO into the oxidized form of GSH and ammonia (Petřivalský et al., 2015). GSNOR is considered as a key regulator of plant development and is induced in response to stresses (Leterrier et al., 2011; Kubienová et al., 2014). Increased activity of GSNOR was observed as a plant reaction to various abiotic stresses, e.g., cold or high temperature (Kubienová et al., 2014; Petřivalský et al., 2015; Tichá et al., 2016). However, it was difficult to find a general tendency in modulation of GSNOR activity in plants exposed to a distinct type of abiotic stressors, e.g., cadmium or arsenate decreased gene expression and GSNOR activity (Petřivalský et al., 2015). Lindermayr (2018) proposed GSNOR as a main component of the cross-talk between ROS and NO. According to his concept, during oxidative burst GSNOR loses its activity due to a modification of cysteine, leading to an accumulation of GSNO, which initiates NO signaling and induces the cellular antioxidant machinery. Therefore, the additional aim of our study was to investigate GSNOR activity, protein abundance, and gene transcription in roots of tomato plants characterized by CAN-induced secondary oxidative stress accompanied by lower NO production.

The chemical nature of NO and NO-derived molecules suggests that the signal transduction involves post-translational modifications of proteins (PTM), with *S*-nitrosylation and nitration being the most widely studied (Mata-Pérez et al., 2016). Therefore, protein modifications are expected to be the key mechanisms when considering the downstream effects of the NO molecule. Protein nitration is the reaction of nitrating agent with a tyrosine residue of the target proteins and results in formation of stable 3-nitrotyrosine (3-NT) (Kolbert et al., 2017). Formation of 3-NT modifies protein structure leading to changes in protein activity. This PTM, mediated by ONOO^-^, could be considered as a marker of nitro-oxidative stress. As demonstrated earlier, short term (24 h) CAN supplementation of tomato seedlings resulted in an increase in 3-NT concentration in root proteins as compared with plants growing in water and corresponds well to ONOO^-^ formation (Krasuska et al., 2016a). For that reason, a next step of this work was to identify 3-NT-immunopositive proteins that were abundant in roots of CAN-treated plants. We suspected that some of them could be stress-related proteins, elements of the cellular antioxidant system, as they were previously identified in other plant material to be targets of tyrosine nitration (Mata-Pérez et al., 2016).

## Materials and Methods

### Plant Material

Tomato seeds (*Solanum lycopersicum* L. cv. Malinowy Ożarowski) (obtained commercially from PNOS Sp. z o.o.) were germinated in water at 20°C in darkness for 3 days. After this period, seedlings of equal roots’ length (5 mm) were selected and transferred to Petri dishes (ϕ 15 cm) filled with filter paper wetted with water (control) or CAN (L-stereoisomer, Sigma-Aldrich) dissolved in distilled water. CAN at concentrations of 10 and 50 µM (inhibiting root growth in 50 and 100%, respectively) was used according to Krasuska et al. (2016b). Control seedlings and seedlings treated with CAN were cultured in a growth chamber at 23/20°C, 12/12 h day/night regime, and light intensity 150-µmol PAR m^-2^ s^-1^ for 24 or 72 h as described by Krasuska et al. (2016b).

### Measurement of Arginine-Dependent Nitric Oxide Synthase-Like Activity

Arg-dependent NOS-like activity was measured according to a method described by Dawson and Knowles (1999) with modification by Krasuska et al. (2016c). Freshly collected roots were washed in distilled water and homogenized in an ice bath in 50-mM 4-(2-hydroxyethyl)-1-piperazineethanesulfonic acid–potassium hydroxide (HEPES) pH 7.0 containing: 5-mM dithiothreitol (DTT), 300-mM sucrose, 10% (w/v) glycerol, 0.1% (w/v) Triton X-100, 1% (v/v) cocktail of protease inhibitors (Sigma-Aldrich), and 2% (w/v) polyvinylpolypyrrolidone (PVPP). After centrifugation at 13,000*g* for 15 min at 4°C, the supernatant was desalted using protein concentrator PES, 3K MWCO (Thermo Scientific^™^).

After desalting, supernatants were collected for further analyses. The reaction mixture contained: 50-mM HEPES pH 7.5 with 1-mM nicotinamide adenine dinucleotide phosphate (NADPH), 10-μM DTT, 100-nM calmodulin, 10-mM magnesium chloride (MgCl_2_), 10-Mm calcium chloride (CaCl_2_), 20-μM oxyhemoglobin, 25-μM flavin adenine dinucleotide (FAD), 10-μM flavin mononucleotide (FMN), 10-μM tetrahydrobiopterin, 1-mM ascorbic acid, and 1-mM Arg in 0.1-M hydrochloric acid (HCl). Oxyhemoglobin was freshly prepared from 1-mM hemoglobin (Sigma-Aldrich, H2625) dissolved in 20-mM potassium phosphate buffer pH 7.0 treated with 5-mM sodium dithionite. Oxyhemoglobin concentration, after desalting with Zeba Spin Desalting Columns, 7K MWCO (Thermo Scientific™) was measured at 415 nm (ε = 131 mM^-1^ cm^-1^).

Extracts were preincubated for 10 min at 37ºC. One portion of the extract was preincubated with equal volume of 12.5-mM N^ω^-nitro-l-arginine methyl ester (L-NAME) in 20% dimethyl sulfoxide (DMSO) and the second one only with 20% DMSO. The reaction was started by adding a reaction mixture. An Arg-dependent NOS-like activity was measured using microplate reader (Sunrise, Tecan) at 401 and 421 nm at 37°C as NO-dependent conversion of oxyhemoglobin to methemoglobin. The activity was calculated as a difference between results obtained for both reaction mixtures and using the results obtained from calmodulin-dependent endothelial nitric oxide synthase (eNOS; Sigma-Aldrich, N1533) reaction carried out as described for the plant enzymatic extracts. Arg-dependent NOS-like activity was presented as nanomole NO per minute per gram protein, based on the activity of eNOS (Sigma-Aldrich, N1533). The activity of eNOS was 0.23 nmol NO min^-1^ mg^-1^ protein.

### Measurement of Total Antioxidant Capacity

Total antioxidant capacity in roots was determined by reduction of 2,2-diphenyl-1-picrylhydrazyl (DPPH) (Molyneux, 2004). Roots (0.1 g) were homogenized in 0.5 ml of 80% (v/v) methanol and incubated in an ultrasonic bath for 5 min at 4°C. Homogenates were centrifuged at 7,000*g* for 10 min at 4°C. After homogenization, 2 µl of the extract was added to 298 μl of 60-μM DPPH dissolved in methanol. The reaction mixture was incubated for 15 min in darkness at room temperature. Concentration of reduced DPPH was measured at 517 nm using microplate reader (Sunrise, Tecan). Antioxidant capacity was expressed as reduction of DPPH defined as [(A_0_-A_s_)/A_0_] x 100%, where A_0_ is absorbance of a blank, and A_s_ is absorbance of the sample.

### Measurement of Total Thiol Content

The total thiol content in roots was measured using Ellman’s reagent (Chan and Wasserman, 1993). Roots (0.2 g) were washed with distilled water and homogenized in an ice bath in 0.5 ml of 0.1-M potassium phosphate buffer (pH 7.5) with 2-mM DTT, 10% (w/v) glycerol, 0.1% (w/v) triton X-100, 1% (v/v) cocktail of protease inhibitors (Sigma-Aldrich), and 2% (w/v) PVPP. Homogenates were centrifuged at 13,000*g* for 15 min at 4°C. Thiol content assay was performed in the mixture: 50 µl of the supernatant, 50 µl of 5,5’-dithiobis (2-nitrobenzoic acid) (DNTB), and 200 µl of 0.1-M potassium phosphate buffer (pH 7.5). The mixture was incubated for 10 min in the darkness at room temperature. The total thiol content was measured at 412 nm using microplate reader (Sunrise, Tecan). The concentration of 2-nitro-5-thiobenzoate (TNB) was calculated using the extinction coefficient ε = 14.15 mM^-1^ cm^-1^. The concentration was expressed as micromole TNB per gram fresh weight (FW).

### Determination of the Enzymatic Activity of the Cellular Antioxidant System

#### Preparation of the Protein Extract for Catalase and Superoxide Dismutase Activity Determination

Roots (0.2 g) were homogenized in an ice bath in 0.1-M potassium phosphate buffer (pH 7.2) with 1-mM ethylenediaminetetraacetic acid (EDTA), 5% (w/v) glycerol, 5-mM DTT, 1% (v/v) cocktail of protease inhibitors (Sigma-Aldrich), and 2% (w/v) PVPP. After centrifugation at 13,000*g* for 15 min at 4°C, supernatant was collected and used for further analysis.

#### Measurement of Catalase Activity in the Polyacrylamide Gel Under Non-Denaturing Conditions

CAT activity was analyzed in a gel according to Woodbury et al. (1971). Soluble protein (10 μg) were separated using 8% polyacrylamide gel electrophoresis under non-denaturing and non-reducing conditions at 4°C. Visualization of CAT activity was performed with 0.003% (v/v) H_2_O_2_ (20 min in darkness); next, the gels were washed in distilled water and stained with the mixture of (1:1) 2% (w/v) iron(III) chloride and 2% (w/v) potassium ferricyanide.

#### Measurement of Superoxide Dismutase Activity in the Polyacrylamide Gel Under Non-Denaturing Conditions

The activity of SOD was analyzed in the gel according to Kuo et al. (2013). Soluble protein (20 μg) samples were subjected to 10% polyacrylamide gel and electrophoretically separated under non-denaturing and non-reducing conditions at 4°C. After an electrophoresis, the gel was incubated in 0.1% (w/v) Nitro blue tetrazolium chloride (NBT) and then in riboflavin solution (28-μM riboflavin and 28-mM N,N,N’,N-tetramethylethylenediamine) in 0.1-M potassium phosphate buffer, (pH 7.4). The visualization of the bands was done by exposure of the gel to UV light for 10 min.

To distinguish SOD isoforms, we used potassium cyanide, which inhibits CuZnSOD activity and H_2_O_2_, which inhibits both CuZnSOD and FeSOD activities.

#### Measurement of Superoxide Dismutase Activity in Extracts of Tomato Roots

SOD activity in extracts of roots was determined with epinephrine assay (Misra and Fridovich, 1972). Roots (0.1 g) were washed with distilled water and homogenized in an ice bath in 0.5 ml of 0.1-M Tris-HCl buffer (pH 7.0) with 5-mM DTT, 0.1% (w/v) sodium deoxycholate, 1% (v/v) cocktail of protease inhibitors (Sigma-Aldrich), and 2% (w/v) PVPP. Homogenates were centrifuged at 13,000*g* for 15 min at 4°C. SOD activity was measured in a mixture: 10, 15, or 20 µl of the supernatant, 10 µl of freshly prepared 10-mM epinephrine in 10-mM HCl, and an appropriate volume of 0.1-M glycine buffer (pH 10) containing 0.1-M sodium chloride (NaCl). Total volume of the assay mixture was 300 µl. Adrenochrome content was measured at 480 nm using microplate reader (Sunrise, Tecan). The amount of extract required for 50% inhibition of the oxidation of the epinephrine was taken for the calculation of SOD activity. Results were compared with a standard curve for SOD activity prepared using a commercial SOD (Sigma-Aldrich, S7571-30KU). The activity was expressed as units per milligram protein.

#### Preparation of Enzymatic Extracts for Glutathione Reductase and Glutathione Peroxidase Activity Determination

Roots (0.15 g) were homogenized in an ice bath in 0.5 ml of 0.1-M potassium phosphate buffer (pH 7.0) with 5-mM DTT, 1% (v/v) cocktail of protease inhibitors (Sigma-Aldrich), and 2% (w/v) PVPP. After centrifugation at 13,000*g* for 15 min at 4°C, supernatant was desalted using protein concentrator PES, 3K MWCO (Termo Scientific^™^).

#### Glutathione Reductase

A measurement of GR activity was performed according to Esterbauer and Grill (1978). An enzymatic extract (25 µl) was incubated in a 200-µl reaction mixture [0.05-M potassium phosphate buffer (pH 7.0) with 0.625-mM oxidized form of glutathione (GSSG) (Sigma-Aldrich)] in the dark for 10 min at room temperature. The measurement of GR activity was started by 25 µl of 2-mM NADPH. The GR activity was measured as a decrease of absorbance at 340 nm, using microplate reader (Sunrise, Tecan). The activity was expressed as units per milligram protein. Unit (U) was defined as nanomoles of substrate utilized by the enzyme in 1 min.

#### Glutathione Peroxidase

A measurement of GPx activity was performed according to Flohé and Günzler (1984) with some modifications (Fontaine et al., 1994) as described by Krasuska and Gniazdowska (2012). An enzymatic extract (25 µl) was incubated with a 0.2-ml reaction mixture: 0.05-M potassium phosphate buffer (pH 7.0) with 0.1-M aminotriazole, 2.5-mM EDTA, 1.25-mM GSH, and 1.5 U of GR (Sigma-Aldrich, G3664) at 25°C for 10 min. After incubation, 50 µl of 2-mM H_2_O_2_ was added. The reaction was started by adding 25 µl of 2.5-mM NADPH. The GPx activity was determined as an absorbance decrease monitored at 340 nm using a microplate reader (Sunrise, Tecan). The activity was expressed as nanomoles NADPH per minute per microgram protein.

### Catalase, Superoxide Dismutase, Glutathione Reductase, Glutathione Peroxidase, and Nitrosoglutathione Reductase Gene Expression Analysis

The expression of genes was assessed in roots using quantitative real-time polymerase chain reaction (qRT-PCR). Total RNA was extracted and purified using an RNAzol RT (Sigma-Aldrich) according to manufacturer’s instructions. RNA samples were DNase treated with DNase I (Thermo Scientific^™^). Total RNA (200 ng) was used to generate first-strand complementary DNA (cDNA) by RevertAid First Strand cDNA Synthesis Kit (Thermo Scientific^™^) with oligo(dT)18 Primer in a total volume of 35 μl, as is described in the manufacturer’s guideline. qRT-PCR was performed in a CFX Connect^™^ Real-Time PCR System. iTaq^™^ Universal SYBR^®^ Green Supermix (Bio-Rad) was used as the basis for the reaction in a total volume of 12 μl (6-μl PCR Supermix, 1-μl primer, 4-μl H_2_O, and 1-μl cDNA).

**Table S1** shows the primer pairs used to amplify the genes.

For the normalization of the expression levels, housekeeping genes *EF1*α and *PP2Acs* were used as a reference genes; cDNA from untreated material was used as a reference sample.

### Immunofluorescence Nitrosoglutathione Localization in Root Axis of Tomato Seedlings

Fragments of root tips (0.3 cm) were immediately fixed in 4% (w/v) paraformaldehyde in 0.1-M microtubule stabilizing buffer (pH 6.9) with 0.1% (w/v) Triton X-100 for 2 h at room temperature as described by Gubler (1989). Samples were dehydrated in ethanol with 10-mM DTT and infiltrated in a mixture of butyl–methyl–methacrylate (BMM) resin with ethanol in dilutions: 1:3, 1:1, and 3:1, and finally in a pure BMM. Polymerization was done for 20 h at -20°C. Acetone was used to remove the BMM from 2.0-μm sections collected on silane-coated slides (Thermo Scientific^™^). An immunofluorescence analysis was carried out after preincubation in 3% (w/v) bovine serum albumin in phosphate-buffered saline (PBS) (3.2-mM sodium hydrogen phosphate, 0.5-mM monopotassium phosphate, 135-mM NaCl, 1.3-mM potassium chloride, pH 7.2) for 1 h at room temperature. Sections were incubated with primary rat anti-GSNO antibodies (Agrisera, AS08 361) in PBS buffer (pH 7.2) (dilution 1:500) for 2 h at room temperature in a humid chamber. Controls for background staining were performed by replacing the primary antibody with the incubation buffer (**Figure S1**). Slides washed with PBS with Tween 20 buffer were treated at room temperature in the dark for 2 h with secondary goat anti-rat immunoglobulin G conjugated to TexasRed-X (Thermo Scientific^™^, T-6392) in PBS buffer (dilution 1:500). An Olympus AX70 Provis (Olympus Poland) with a UM61002 filter set and equipped with an Olympus SC35 camera was used for fluorescence imaging.

### Nitrosoglutathione Reductase Activity Assay in Extracts of Tomato Roots

Activity of GSNOR was measured according to Sakamoto et al. (2002) with some modifications by Krasuska et al. (2017). Root were homogenized in an ice bath with 50-mM Tris-HCl pH 8.0, 1-mM EDTA, 5% (w/v) glycerol, 0.1-mM phenylmethylsulfonyl fluoride (PMSF), 5-mM DTT, 5-mM MgCl_2_, 1% (v/v) protease inhibitor cocktail (Sigma-Aldrich), and 2% (w/v) PVPP centrifuged at 10,000*g* 10 min. A supernatant was concentrated and desalted using concentrator PES, 3K MWCO (Thermo Scientific^™^) at 10,000*g* for 30 min. A reaction mixture (300 µl) contained: 8 µg of protein extract, 0.5-mM EDTA, 0.2-mM nicotinamide adenine dinucleotide (reduced form) (NADH) in 50-mM Tris-HCl pH 8.0. The reaction was initiated by addition of GSNO to the reaction mixture at a final concentration of 0.6 mM. A GSNOR activity was measured as absorbance decrease at 340 nm for 6 min, using a microplate reader (Sunrise, Tecan). The activity was calculated using the extinction coefficient ε = 6.22 mM^-1^ cm^-1^ and expressed as micromole NADH per gram FW.

### Nitrosoglutathione Reductase Activity Detection by Staining Following Native Gel Electrophoresis

Detection of the GSNOR activity in the gel was done according to Kubienová et al. (2014) with some modifications as described by Krasuska et al. (2017). Roots (0.2 g) were homogenized in 0.5-ml extraction buffer 50-mM Tris-HCl pH 7.5, 0.2% (w/v) Triton X-100, 1% (v/v) protease inhibitor cocktail (Sigma-Aldrich), 5% (w/v) glycerol, and 2% (w/v) PVPP. Extracts were centrifuged for 10 min at 10,000*g* and desalted using protein concentrators PES, 3K MWCO (Termo Scientific^™^). Samples of 30 µl containing 50-µg proteins were mixed with 10 µl of 60% glycerol and loaded for electrophoretic separation using 10% native polyacrylamide gels. Then, gels were rinsed in deionized water, placed in the mixture: 0.1-M sodium phosphate buffer, pH 7.4, 2-mM NADH for 15 min. Next, two filter papers soaked with 4-mM GSNO in 0.1-M sodium phosphate buffer pH 7.4 were placed on the gel and incubated in darkness for 15 min. Detection of GSNOR activity in the gel was done after UV excitation.

### Detection of Nitrosoglutathione Reductase Protein Level

Detection of GSNOR protein was done by immunoblotting as was described by Kubienová et al. (2016). Roots were homogenized in 0.1-M Tris-HCl, pH 7.5 with 1-mM EDTA, 2% (w/v) PVPP, 1-mM DTT, 1% (v/v) protease inhibitor cocktail (Sigma-Aldrich), 0.1% (w/v) Triton X-100, 1-mM MgCl_2_, and 10% (w/v) glycerol in an ice bath. After centrifugation at 10,000*g* for 15 min at 4°C, the supernatant was collected for further analysis. Protein samples were suspended in the sample buffer: 63-mM Tris-HCl, pH 6.8, 1% (w/v) sodium dodecyl sulfate (SDS), 10% (w/v) glycerol, 0.01% (w/v) bromophenol blue, and 20 mM DTT. After incubation at 95°C for 5 min, 7.5-µg proteins were loaded per lane and separated on 10% polyacrylamide gels with SDS (Laemmli, 1970) and then electrotransferred to nitrocellulose membranes (Pure Nitrocellulose Membrane, Sigma-Aldrich) using a Bio-Rad wet blotting apparatus (Towbin et al., 1979). The membranes were blocked overnight at 4°C with nonfat dry milk in Tris-buffered saline and Tween 20 (TBST). After blocking, membranes were washed three times in TBST and immunolabeled with anti-GSNOR polyclonal rabbit antibodies diluted 1:1,000 (Kubienová et al., 2013). As secondary antibodies, anti-rabbit immunoglobulin G conjugated with alkaline phosphatase (Sigma-Aldrich, A3687) at a dilution of 1:100,000 at room temperature was used. Visualization of GSNOR band was done after addition of 0.1-M Tris-HCl pH 9.5, 0.1-M NaCl, 5-mM MgCl_2_, 0.2-mM NBT, and 0.21-mM 5-bromo-4-chloro-3-indolyl phosphate (BCIP).

### Mass Spectrometry Analyses of Proteins

#### Protein Extraction and Purification

Roots (2 g) were homogenized in liquid nitrogen, and then, proteins were extracted by adding 5 ml of 0.1-M Tris-HCl buffer (pH 7.0) with 1% (w/v) Triton X-100, 2% (w/v) glycerol, 2-mM DTT, 0.15-M NaCl, 1% (v/v) protease inhibitor cocktail (Sigma-Aldrich), and 5% (w/v) PVPP. After centrifugation, supernatant was concentrated with Pierce^™^ Protein Concentrator PES, 3K MWCO (Thermo Scientific^™^) to obtain 2 ml of protein extract. Protein concentration was measured and equalized in all samples. Monoclonal anti-nitrotyrosine (Agrisera, AS10 706-100) antibodies were added to protein samples (2 µl of the antibodies for each 1 mg of proteins) and mixed gently at 4°C overnight. Then, 5-µl protein G agarose (Thermo Scientific^™^) was added and incubated for 8 h at 4°C with gentle mixing. After centrifugation for 2 min at 2,500*g* at 4°C, supernatant was discarded, and the pellet was dissolved in TBS (0.1 M Tris-HCl with 0.15-M NaCl, pH 8.0) and kept overnight in 4°C. Mixture was centrifuged for 3 min at 2,500 *g* at 4°C, supernatant was discarded, and pellet was suspended in sample buffer [63-mM Tris-HCl, pH 6.8, 1% (w/v) SDS, 10% (w/v) glycerol, 0.01% (w/v) bromophenol blue, and 20 mM DTT] and denatured for 10 min in 95°C. Then, proteins were separated on two 10% polyacrylamide gels with SDS according to Laemmli (1970). One gel was stained with Coomassie, and the second was electrotransferred to nitrocellulose membranes (Pure Nitrocellulose Membrane, Sigma-Aldrich) according to Towbin et al. (1979) using a Bio-Rad wet blotting apparatus. The membrane was blocked overnight at 4°C with nonfat dry milk in TBST. After blocking, membranes were washed three times in TBST, and immunolabeling of 3-NT was carried out by incubating the membranes with monoclonal anti-3-NT antibody (Agrisera, AS10 706-100), conjugated with alkaline phosphatase, at a dilution of 1:100,000 at room temperature. Visualization of proteins containing 3-NT was done after addition of 0.1-M Tris-HCl pH 9.5, 0.1-M NaCl, 5-mM MgCl_2_, 0.2-mM NBT, and 0.21 mM BCIP

#### In-Gel Digestion

Protein bands of nitrated proteins that differentiate between control and CAN treated were cut out (**Figure S2**). Destaining, trypsin digestion, and peptide extraction were done as was described by Eckermann et al. (2002). Gel pieces were washed in a mixture of 40% (v/v) acetonitrile and 60% (v/v) 50-mM ammonium bicarbonate for 1–2 h. Destained gel bands were dried by vacuum centrifugation, and modified trypsin (a sequencing grade, Roche) (30 ng µl^-1^), dissolved in 50-mM ammonium bicarbonate was added. Trypsin digestion was performed at 37°C (overnight). The gel was incubated in sequence: 1) water, 2) acetonitrile, 3) 5% (v/v) formic acid, and again 4) acetonitrile. After each step, supernatants were collected. The combined supernatant was lyophilized and resolved in a mixture of 10% (v/v) acetonitrile and 90% of 0.1% trifluoroacetic acid.

#### Matrix-Assisted Laser Desorption/Ionization Analyses

Tandem mass spectrometry (MS/MS) analysis of extracted proteins was performed with a Thermo LTQ XL, as a matrix served α-cyano-4-hydroxy cinnamic acid [5 mg ml^-1^, dissolved in 84% (v/v) acetonitrile]. For an identified protein, at least 2 peptides were confirmed by MS/MS analysis (**Table S2**). A database search was performed using the MASCOT search engine (Matrix-Science). Peptide tolerance was ±1.2 Da and MS/MS tolerance 0.5 Da.

### Protein Concentration Measurement

Protein content determination was performed using Bradford reagent (Bradford, 1976). As a standard, fatty acid-free bovine serum albumin was used.

### Densitometry Analysis

Densitometry analysis was done using Image J.

### Statistics

All data were obtained in at least three independent experiments with at least two repetitions each. Data were analyzed using Statistica Software. Mean differences were calculated using t-test; standard deviation (SD) was also provided to indicate the variations associated with the particular mean values.

## Results

### Canavanine Inhibited Arginine-Dependent Nitric Oxide Synthase-Like Activity in Roots of Tomato Seedlings

An Arg-dependent NOS-like activity in extracts of roots of control tomato seedlings did not differ during the culture period and was about 0.33–0.38-nmol NO min^-1^ g^-1^ protein (**Table 1**). After a short-term supplementation, an Arg-dependent NOS-like activity was inhibited by 60% in 10-µM CAN and by 70% in 50-µM CAN. After extending the experiment for an additional 48 h, 10-µM CAN inhibited Arg-dependent NOS-like activity in root extracts by around 50%, while 50-µM CAN by more than 70% (**Table 1**).

**Table 1:** Arg-dependent NOS-like activity in extracts from roots of the control plants grown in water or from roots of tomato seedlings treated with CAN (10 or 50 µM) for 24 and 72 h.

Plant treatment	Arg-dependent	
	NOS-like activity (nmol NO min^-1^ g^-1^ protein)	
	24 h	72 h
Control (water)	0.33 ± 0.05	0.38 ± 0.01
CAN 10 µM	0.12 ± 0.02*	0.20 ± 0.03*
CAN 50 µM	0.09 ± 0.01*	0.10 ± 0.01*

Values are average ± SD of three to four repetitions. Asterisks (*) indicate significance between treatments and the control at the same time of culture period at P ≤ 0.05, based on Student’s test.

### Canavanine Increased Total Antioxidant Capacity and Level of Total Thiols in Extracts of Tomato Roots

The total antioxidant capacity in root extracts of control plants after 24 h of culture was about 41% reduction of DPPH and increased to 57% as the experiment was prolonged (**Table 2**). Short-term treatment of seedlings with 50-µM CAN enhanced total antioxidant capacity to 64%, while CAN at lower concentration had no effect on this parameter. As the culture period was extended, antioxidant capacity of root extracts of CAN-exposed plants increased as compared with that of the control.

**Table 2:** Total antioxidant capacity and concentration of total thiols in extracts from roots of the control plants growing in water or from roots of tomato seedlings treated with CAN (10 or 50 µM) for 24 and 72 h.

Plant treatment	Total antioxidant capacity		Total thiol content	
	**(% of DPPH reduction)**		**(µmol TNB g^-1^ FW)**	
	**24 h**	**72 h**	**24 h**	**72 h**

Control (water)	41.2 ± 8.0	57.1 ± 9.5		0.15 ± 0.02	0.16 ± 0.03
CAN 10 µM	36.6 ± 5.5	65.9 ± 11.2		0.29 ± 0.04*	0.33 ± 0.05*
CAN 50 µM	64.2 ± 4.3*	87.4 ± 5.1*		0.21 ± 0.02*	0.38 ± 0.03*

Values are average ± SD of three to four repetitions. Asterisks (*) indicate significance between treatments and the control at the same time of culture period at P ≤ 0.05, based on Student’s test.

The content of total thiols in roots of the control plants did not differ during the culture period and was 0.15-µmol TNB g^-1^ FW (**Table 2**). A supplementation with CAN for 24 h resulted in 40% increase (after 50-µM CAN) and almost doubled the amount of total thiols after exposition to 10 µM CAN. Prolonged treatment with CAN led to further accumulation of total thiols; their level was more than twice higher in the roots of treated plants compared with that in the control (**Table 2**).

### Activity of Enzymatic Antioxidant System Was Inhibited by Canavanine

**Figure 2:**
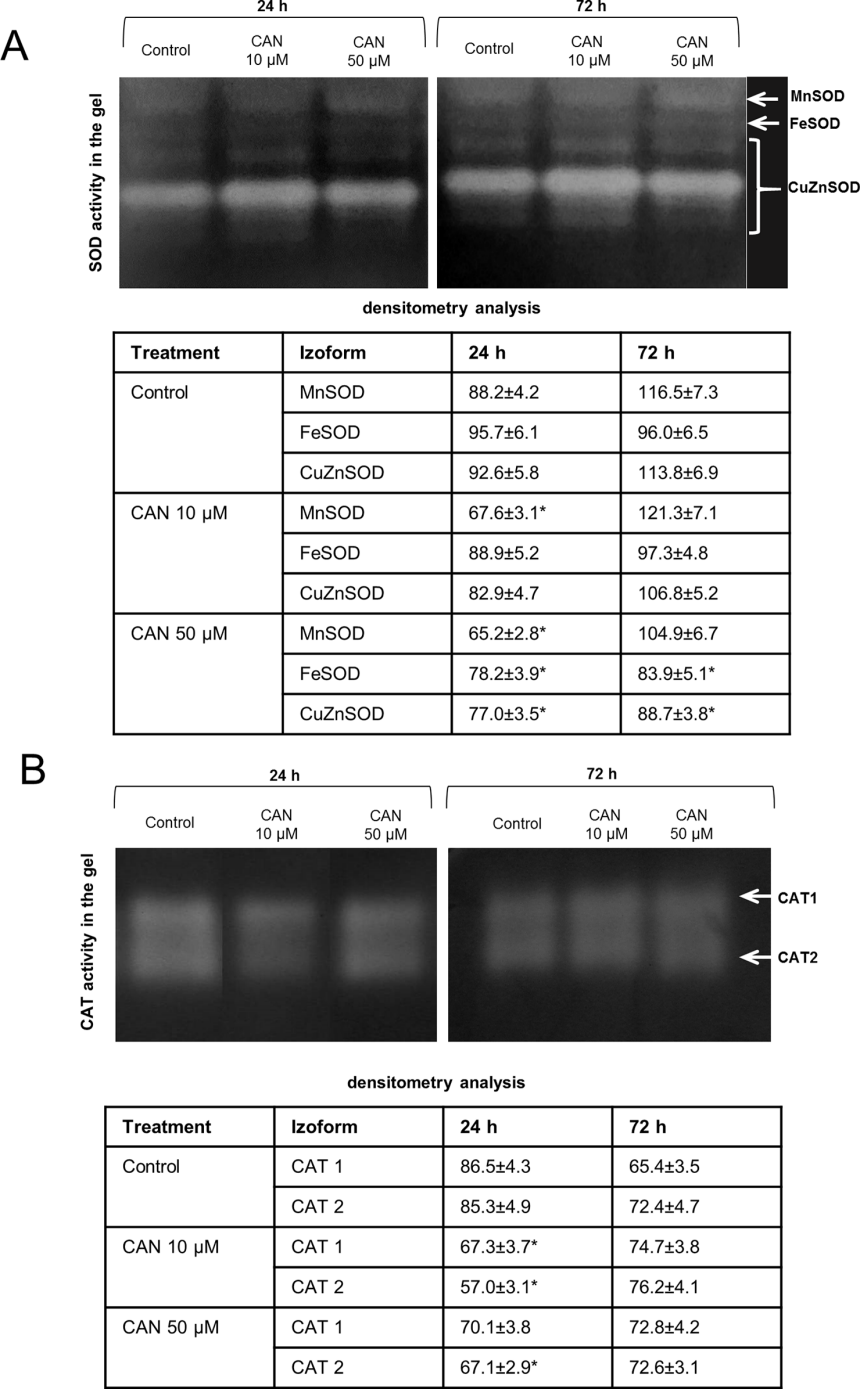
In-gel detection of SOD **(A)** and CAT **(B)** activities in extracts from roots of the control seedlings growing in water and roots of seedlings treated with CAN (10 or 50 µM) for 24 and 72 h. Total soluble proteins (20 µg per lane for SOD activity measurement and 10 µg per lane for CAT activity measurement) were electrophoretically separated using 10% (for SOD) and 8% (for CAT) gels under non-denaturing and non-reducing conditions. For visualization of SOD activity gels were incubated in 0.1% (w/v) NBT and then in riboflavin solution, for visualization of CAT activity gels were stained with the mixture of (1:1) 2% iron(III) chloride and 2% potassium ferricyanide. For SOD activity, visualization was done by gel exposure to UV light for 10 min. SOD and CAT isoforms are marked by arrows. Experiments were performed three times, and representative data are shown.

CAN declined **SOD activity** both measured in-gel (**Figure 2A**) and by spectrophotometric method (**Figure S3**). In control and CAN-supplemented roots, the strongest was CuZnSOD isoform, while MnSOD and FeSOD were weaker (**Figure 2A**). Activity of CuZnSOD in roots treated with 50-µM CAN was 15 and 23% lower than that in the control after 24 and 72 h, respectively (**Figure 2A**). MnSOD was inhibited in CAN-exposed roots in about 25% after 24 h, whereas after additional 48 h, bands corresponding to MnSOD activity were slightly brighter, showing less dissimilarity in all combinations (**Figure 2A**). Inhibition by CAN of total activity of SOD was more spectacular in the spectrophotometric assay (**Figure S3**). SOD activity in roots exposed to 10-µM CAN was reduced by 24 and 39% after 24 and 72 h, respectively. CAN at higher concentration inhibited SOD activity in tomato roots by 39% after 24 h and 60% after an additional 48 h (**Figure S3**).

In-gel **CAT activity** analysis indicated two CAT isoforms present in tomato roots extracts (**Figure 2B**). The highest CAT activity was noticed for the control plants after 24 h of the culture. Less visible two bands of CAT activity have been seen after 24 h of 10-µM CAN supplementation. The culture of tomato seedlings for 24 h in 50-µM CAN decreased CAT2 activity. No statistically significant differences in CAT activity were noticed after 72 h of CAN treatment (**Figure 2B**).

**Figure 3:**
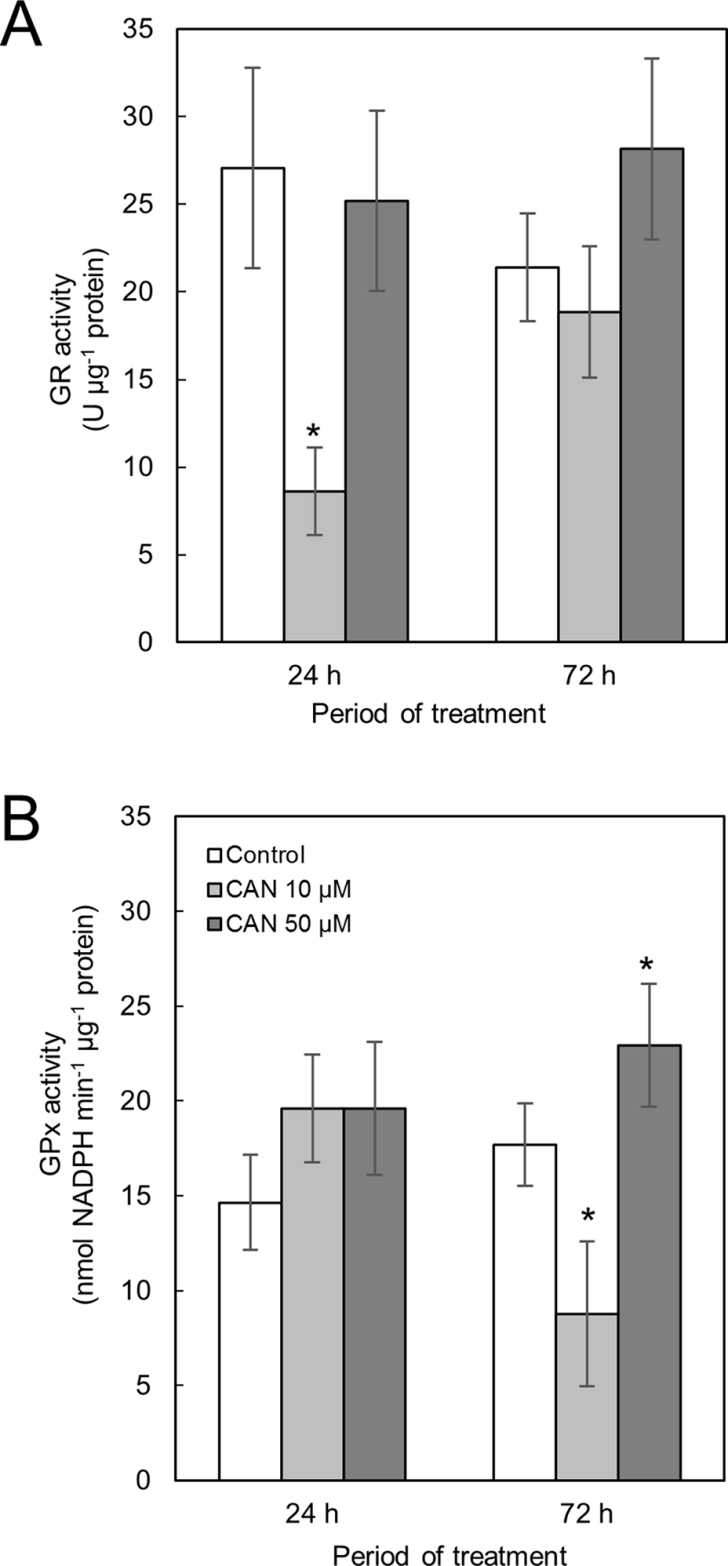
Activity of GR **(A)** and GPx **(B)** in extracts from roots of the control seedlings growing in water and roots of seedlings treated with CAN (10 or 50 µM) for 24 and 72 h. Values are average ± SD of at least three independent experiments and three biological repetitions each. Asterisks (*) indicate significance between treatments and the control at the same time of culture period at *P* ≤ 0.05, based on Student’s test.

**Activity of GR** in control tomato roots was stable during the culture period (**Figure 3A**). Short-term exposure of the seedlings to CAN in low concentration resulted in a drastic reduction of GR activity, while after additional 48 h, GR activity in roots of these plants increased twice and was at the level of the control. CAN at higher (50 µM) concentration had no effect on GR activity after 24 h supplementation. After 72-h treatment with 50-µM CAN, activity of GR was similar as after 24 h and insignificantly higher than that in the control (**Figure 3A**).

**Activity of GPx** in roots of control plants increased less than 20% during the experiment (**Figure 3B**). Short-term treatment with CAN at both concentration slightly stimulated GPx activity. Prolonged supplementation of tomato seedlings with 10-µM CAN resulted in drastic drop in GPx activity in roots, while CAN at higher concentration (50 µM) led to further stimulation of GPx activity (**Figure 3B**).

### Canavanine Modified Expression of Genes Coding Antioxidant Enzymes

**Figure 4:**
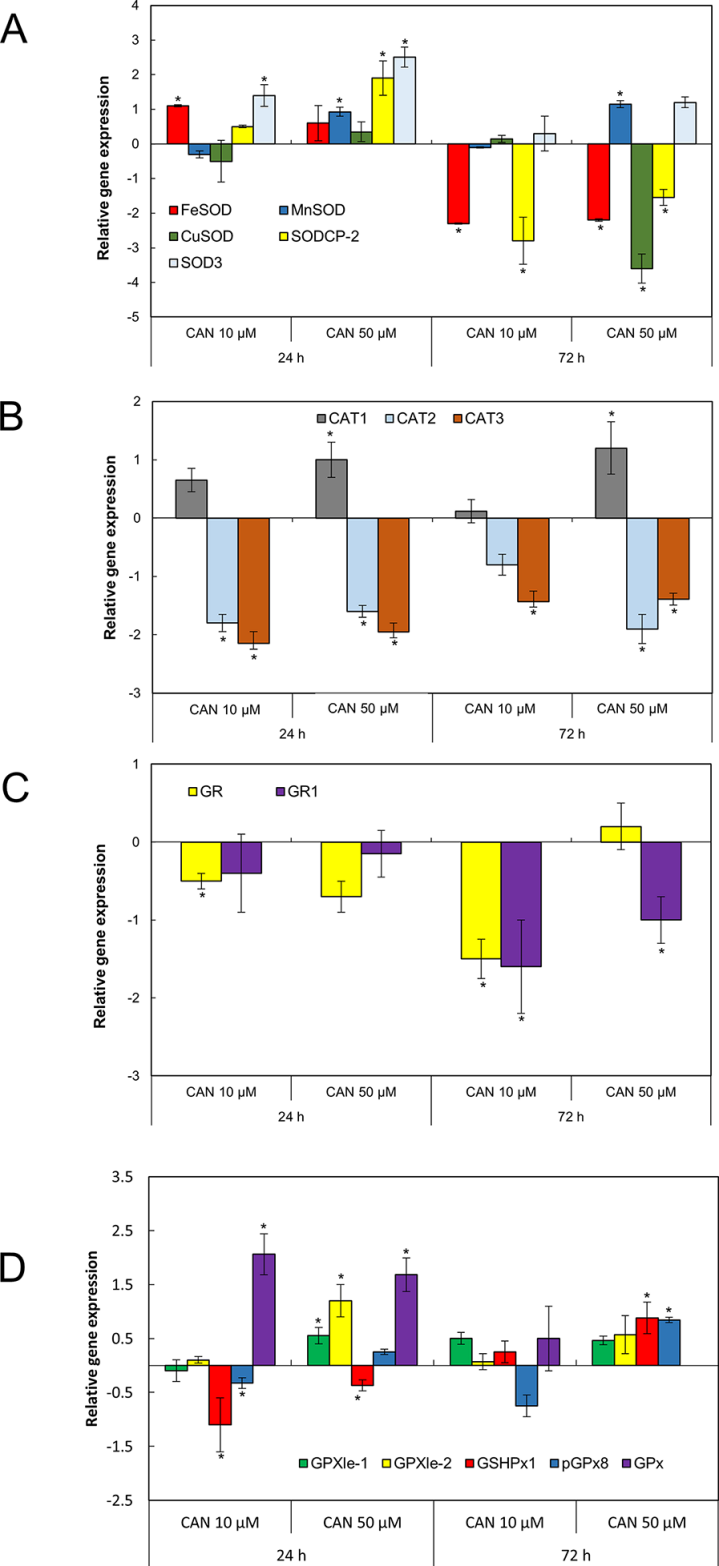
The expression level of genes encoding different isoforms of SOD: *MNSOD*, *CUSOD2*, *FESOD*, *SODCP.2*, *SOD3* **(A)**; CAT: *CAT1*, *CAT2*, *CAT3* **(B)**; GR: *GR*, *GR1* **(C)**; and GPx: *GPXle-1*, *GPXle-2*, *GSHPx1*, *pGPx8*, *GPx* **(D)** analyzed by qRT-PCR as described in the section of *Material and Methods*. Roots of tomato seedlings were collected after 24 or 72 h of CAN (10, 50 µM) treatment; as a control, roots of plants cultured in water were used. Asterisks (*) indicate significance between treatments and the control at the same time of culture period at *P* ≤ 0.05, based on Student’s test.

*SOD* transcript levels in roots was determined for five genes coding three Mn-FeSOD family enzymes (*MNSOD*-ID:101256386, *SOD3*-ID:101256231, and *FESOD*-ID: 544259) and two isomers of CuZnSOD family enzymes (*SODCP.2*-ID: 543981 and *CUSOD2*-ID:101264296), NCBI database (**Figure 4A**). After 24 h of CAN supplementation, *FeSOD*, *SODCP.2*, and *SOD3* were upregulated. In contrast, prolongation of the experiment led to downregulation of *FeSOD* and *SODCP.2* (by CAN at both tested concentration) and downregulation of *CUSOD* by 50-µM CAN (**Figure 4A**). *SOD3* and *MnSOD* were upregulated in roots of plants growing in CAN for 72 h (**Figure 4A**).

Three genes encoding different isoforms of CAT—*CAT1* (ID: 543990), *CAT2* (ID:543585), and *CAT3* (ID: 101259333)—are present in tomato roots. Irrespective of the concentration and duration of the culture, two of the genes, *CAT2* and *CAT3*, were downregulated by CAN (**Figure 4B**). In contrast, *CAT1* was upregulated by 50-µM CAN (**Figure 4B**).

Two genes (*GR* and *GR1*) coding different isoforms of GR are present in tomato roots (**Figure 4C**). Irrespective of the concentration and duration of the culture, both of the genes were downregulated by CAN, with the exception of *GR1*, expression of which was at the level of the control after 24 h of CAN at both concentration and GR for 10-µM CAN after 24 h and 50-µM CAN after 72 h (**Figure 4C**).

In tomato roots, there are five genes coding various isoforms of GPx (**Figure 4D**). After 24 h of the experiment in CAN-stressed plants, *GSHPx1* was downregulated. Twenty-four-hour exposition to CAN (both 10 and 50 µM) resulted in upregulation of *GPx* (**Figure 4D**). Higher transcript level was characteristic also for *GSHPx1* and *pGPx8*, as the 50-µM CAN treatment was prolonged. The exception was *GPx*, expression that was not detected in roots grown in 50-µM CAN for 72 h; for other genes, no significant changes were noted (**Figure 4D**).

### Canavanine Restricted the Accumulation of Nitrosoglutathione in Root Tips

**Figure 5:**
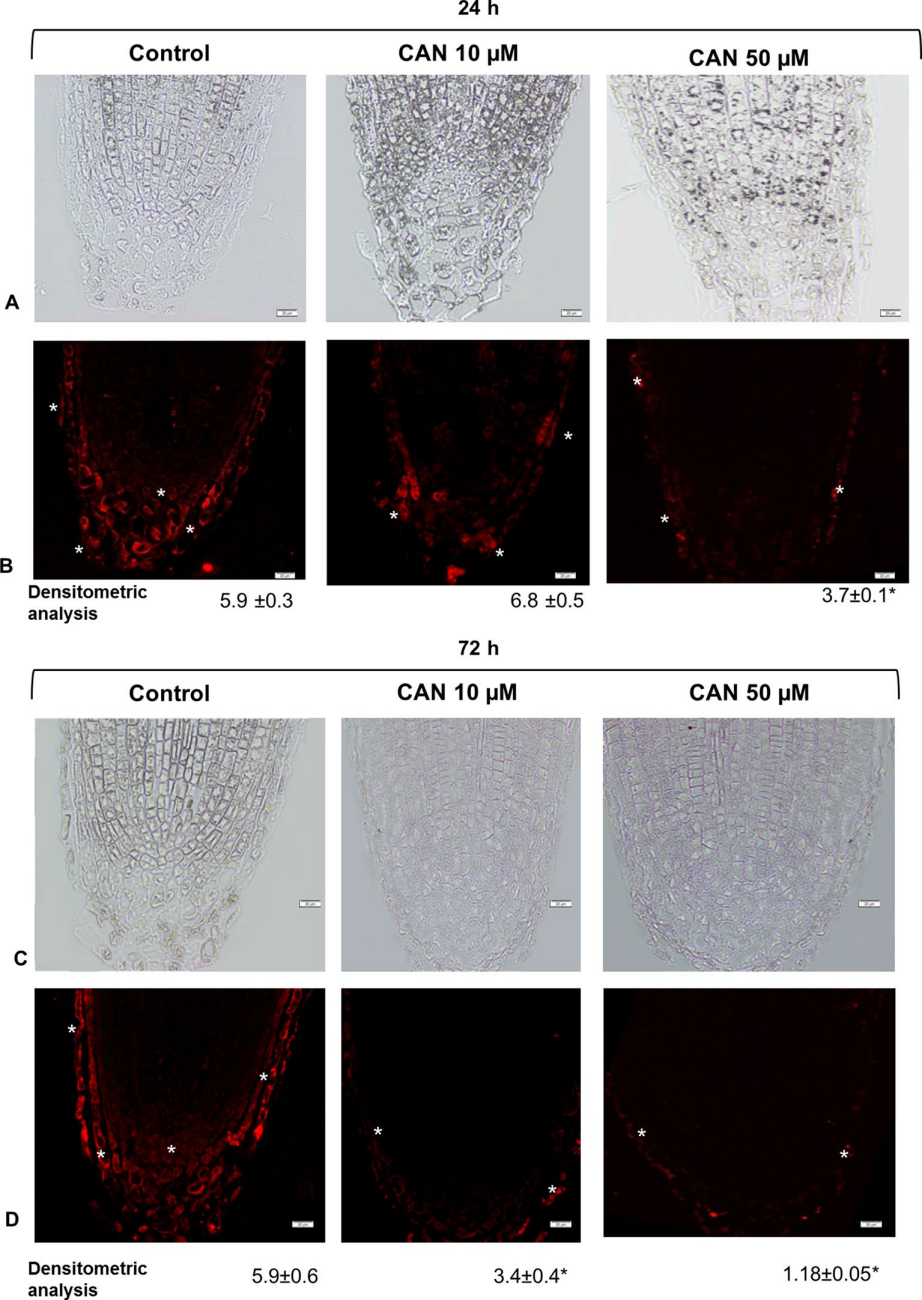
GSNO-related red fluorescence signal (*) in root apex of tomato seedlings growing in CAN (10 or 50 µM) for 24 h (panels **A** and **B**) and 72 h (panels **C** and **D**). Panels **(A)** and **(C)** present the bright field, panel **(B)** and **(D)** corresponding to them fluorescence images. Scale bars 20 µm. Experiments were performed three times, and representative data are shown.

Localization of GSNO on longitudinal sections of the tomato seedlings root apex was investigated by a single immunofluorescence labeling technique. Control roots showed GSNO red fluorescence signal in root caps, predominantly in separated root border cells, but also in promeristem division zone (**Figures 5B, D**). After CAN treatment, the fluorescent signal was reduced as compared with that in control plants (**Figures 5B, D**). Moreover, GSNO was localized mainly in the rhizodermis and root caps (depending on the analyzed root zone). The longer the CAN was supplemented, the weaker the GSNO signal was observed (**Figure 5D**). After exposition of tomato seedlings to 50 µM, CAN for 72-h fluorescence signal in roots was observed almost only in the external part of root cap cells (**Figure 5D**). Additionally, in root apex sections where primary antibodies were omitted, there was no red fluorescence signal noticed (**Figure S1**).

### Canavanine Slightly Modified Nitrosoglutathione Reductase Activity, Enlarged Nitrosoglutathione Reductase Protein Content, and Had No Effect on Nitrosoglutathione Reductase Gene Expression in Tomato Roots

**Figure 6:**
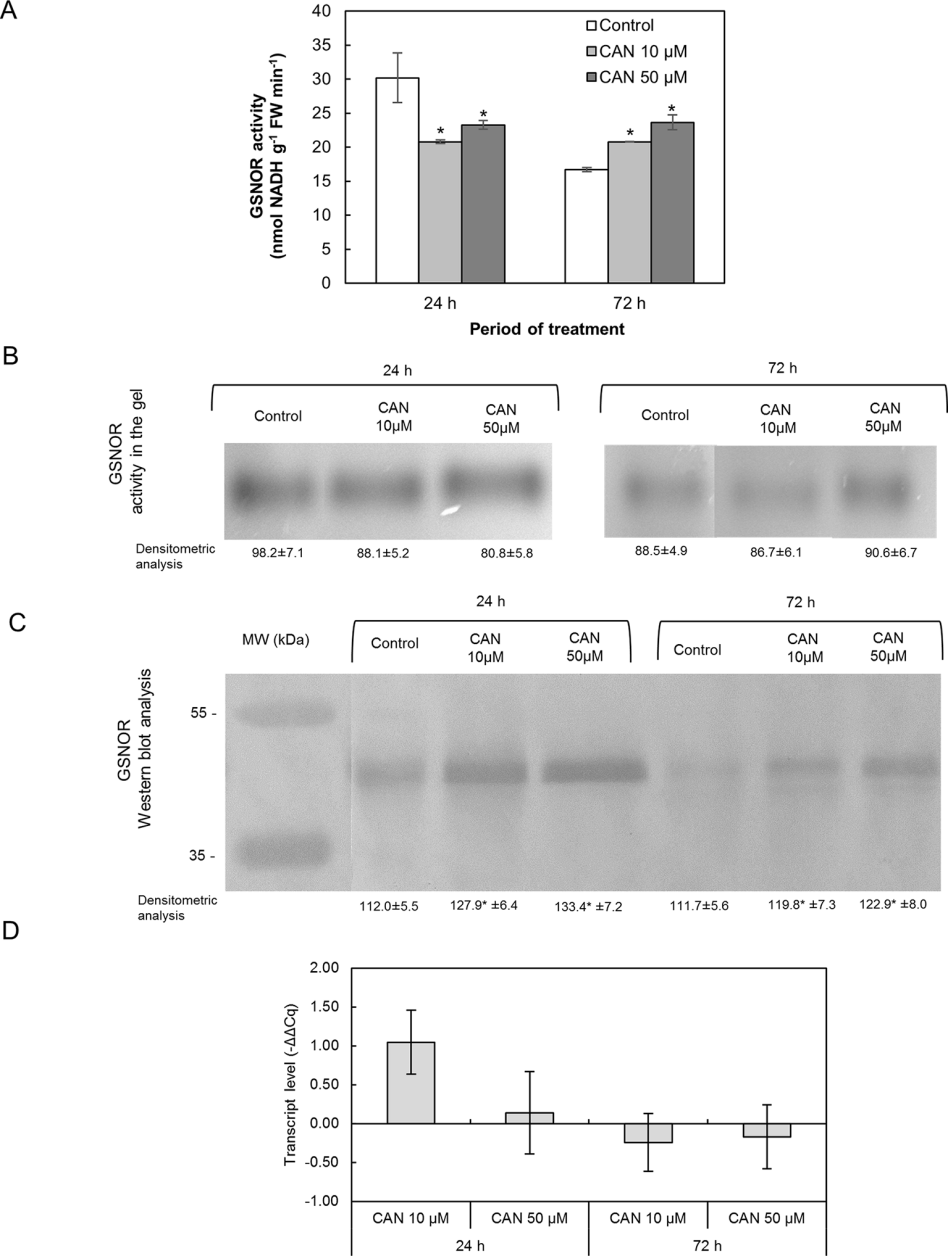
Activity of GSNOR determined in extracts using spectrophotometric assay **(A)**, visualization of GSNOR activity in the gel **(B)**, GSNOR protein **(C)** and transcript level of *GSNOR* **(D)** in roots of tomato seedlings growing in water (control) or treated with CAN (10 or 50 µM) after 24 or 72 h of culture period. Asterisk (*) indicates significance from control at the same time of culture period at *P* ≤ 0.05, based on Student’s test. Values are average ± SD of at least three independent experiments and three biological repetitions each.

Short-term exposition of seedlings to CAN lowered GSNOR activity determined in extracts; it was around 21–23 nmol NADH min^-1^ g^-1^ FW. In extracts of roots of control plants, GSNOR activity declined twice during the culture period (**Figure 6A**). After 72 h of the treatment, GSNOR activity in extracts of tomato roots was at the level observed after 24 h of culture but 25 and 43% higher than in control for 10- and 50-µM CAN, respectively (**Figure 6A**).

The tests on GSNOR activity following native electrophoresis showed no CAN dependence (**Figure 6B**). Bands corresponding to activity of GSNOR were visibly thicker for younger seedlings irrespective of the treatment. Only after 72 h that GSNOR activity staining in the gel showed its enhancement by 50-µM CAN (**Figure 6B**).

Content of GSNOR protein in roots was investigated by Western blot analysis. Single bands corresponding to a protein of molecular weight of about 43 kDa were clearly visible in protein roots’ extracts of the control and CAN-treated seedlings (**Figure 6C**). In extracts from treated plants, bands corresponding to a protein of molecular weight of 43 kDa were thicker than in control irrespective of the duration of CAN application and its concentration though, in general, were more visible in extracts from roots of younger seedlings (after 24 h of culture).

GSNOR gene expression in tomato roots was not affected by CAN supplementation. *GSNOR* expression in CAN treated plants was found to be higher compared to roots of control plants after 24 h of culture in 10 µM CAN (**Figure 6D**).

### Nitrated Proteins in Roots of Canavanine-Stressed Plants Belong to Seed Storage Proteins or Components of the Cellular Redox System

In protein extracts from roots of CAN-supplemented seedlings, we have found several bands differently nitrated in comparison with the control (**Table 3**). In roots of CAN-treated plants irrespective of the duration of the experiment and the concentration of the tested NPAA, phosphoglycerate kinase was identified as nitrated protein. For plants after 24 h of culture, seed storage proteins (11s globulin seed storage of protein 2-like, 12s seed storage of protein CRA1-like, vivilin precursor) and luminal-binding protein 5 were identified as nitration targets. Prolongation of the CAN treatment resulted in nitration of monodehydroascorbate reductase, peroxidase 3-like, and polyphenol oxidase (**Table 3**).

**Table 3:** Matrix-assisted laser desorption/ionization MS/MS identification after trypsin in-gel digestion of the nitrated proteins of tomato roots treated with CAN (10 or 50 µM) for 24 or 72 h.

	Culture period (h)	Description	NBCI ID	Identified peptides (no)
**CAN 10 µM**	24	Luminal-binding protein 5	XP_004234985.1	3
		Phosphoglycerate kinase, chloroplastic	XP_004243968.1	2
		11s globulin seed storage protein 2-like	XP_004247523.1	3
		(fragment)		
		12s seed storage protein CRA1-like	XP_004246943.1	2
		(fragment)		
	72	Polyphenol oxidase D, chloroplastic	NP_001334885.1	4
		Prohibitin-3 mitochondrial	XP_004250114.1	3
		Monodehydroascorbate reductase	NP_001318117.1	4
		Phospoglycerate kinase, chloroplastic	XP_004243968.1	2
**CAN 50 µM**	24	Aconitate hydratase, cytoplasmic	XP_004251517.2	3
		Luminal-binding protein 5	XP_004234985.1	2
		Phosphoglycerate kinase. chloroplastic	XP_004243968.1	2
		Vivilin precursor (fragment)	NP_001308118.1	2
		Prohibitin-1, mitochondrial-like	XP_004251498.1	4
		11s globulin seed storage protein 2-like	XP_004247523.1	3
		(fragment)		
		12s seed storage protein CRA1-like	XP_004246943.1	2
		(fragment)		
	72	Peroxidase 3-like	XP_006367274.1	2
		Polyphenol oxidase D, chloroplastic	NP_001334885.18	3
		Monodehydroascorbate reductase	NP_001318117.1	4
		Phospoglycerate kinase, chloroplastic	XP_004243968.1	3

## Discussion

### Arginine-Dependent Formation of Nitric Oxide Was Inhibited by Canavanine

**Figure 7:**
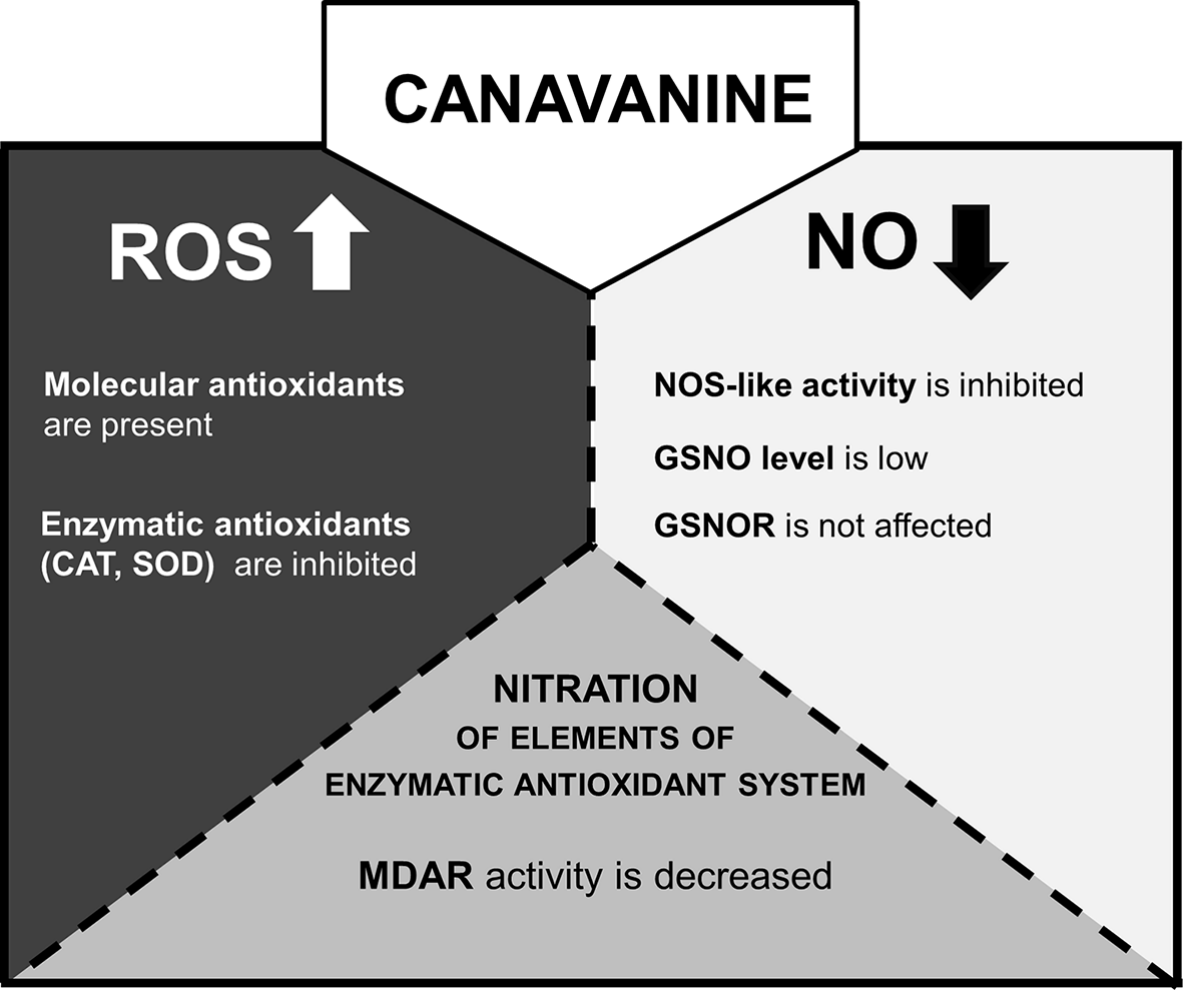
The model of CAN action in tomato roots after prolonged (72 h) supplementation of seedlings with the NPAA. CAN increases level of ROS (Krasuska et al., 2016a) and decreases NO emission (Krasuska et al., 2016a). ROS are accumulated (CAN secondary mode of action) and could inhibit GSNOR activity by oxidative PTMs (Lindermayr, 2018). However, the gene expression and activity of GSNOR are not affected. GSNO level is lowered probably due to limitation of NO resulting from restriction of NOS-like activity (direct mode of action of CAN). ROS over-accumulation is accompanied by stimulation of molecular antioxidant system. CAT or SOD gene expression is downregulated, CAT and SOD enzymatic activities are inhibited. MDAR is an enzymatic antioxidant and the target of differential nitration in CAN-supplemented plants.

Over the last few years, there has been an intense debate about the existence of NOS-like enzyme in the plant kingdom. Based on sequence analysis methods, the main “advocates” of the occurrence of NOS-like protein in plant cells (Jeandroz et al., 2016; Astier et al., 2018) demonstrated that no typical mammalian NOS-like sequences can be found, even in species in which Arg-dependent NOS activity and/or effects of mammalian NOS inhibitors (N^ω^-nitro-L-arginine methyl ester or N^ω^-methyl-l-arginine) have been reported. Although the possibility that plants have NOS protein or adequate protein complex of a structure unrelated to a mammalian type is under consideration, functionality of the reaction was demonstrated in various plant tissue (Corpas and Barroso, 2017). In our previous research, it was shown that CAN, a commonly used inhibitor of mammalian isoform of NO synthase, led to restriction in NO formation in tomato roots (Krasuska et al., 2016a). This observation was the impulse to check CAN impact on one of the putative pathways of NO synthesis in plants. Thus, in the current work, we have clearly confirmed that, in plants, CAN may be used as an inhibitor of Arg-dependent NO formation (**Figure 7**). Prolonged (72 h) supplementation of tomato seedlings with CAN resulted in dose-dependent inhibition of Arg-dependent NOS-like activity. No differences (in comparison with the control) in NO_2_^-^ concentration observed in CAN-supplemented plants (Krasuska et al., 2016b) indicated that CAN does not play an important role in the regulation of reductive pathway of NO biosynthesis. Therefore, low NO emission resulted from CAN application could be explained by demonstrated inhibition of Arg-dependent NO synthesis. Similarly, a negative effect of CAN on NO emission was noticed in apple (*Malus domestica* Borkh.) embryos and was also accompanied by reduced Arg-dependent NOS-like activity (Krasuska et al., 2016c).

### Canavanine Decreased Nitrosoglutathione Level in Root Tips and Had No Effect on Nitrosoglutathione Reductase

A plant reaction to biotic and abiotic stresses involves activation of RNS- and ROS-dependent signaling pathways linked to, e.g., PTMs of proteins. *S*-nitrosylation, which consists of the reversible achievement of NO into cysteine residue, belongs to one of NO-dependent PTMs and led to formation of nitrosothiols (SNOs) (Mur et al., 2013). GSNO is a low molecular SNO and is considered to be one of the main signaling molecules among SNOs and a reservoir of NO. Restriction in NO formation, due to inhibition of Arg-dependent NOS-like activity, in CAN-treated tomato seedlings resulted in decline of the content of GSNO in root tips (**Figure 7**). After prolonged exposition of seedlings to CAN (particularly at higher concentration), GSNO localization was limited only to external part of root tips, which fits well to NO localization (Krasuska et al., 2016b). Similarly, after a high-temperature treatment (Chaki et al., 2011). GSNO was localized in cortex and epidermal cells instead of vascular tissues and suggested that such redistribution of GSNO could be a consequence of the protective mechanisms against stress factors. GSNO moves in vascular tissue and can propagate message about environmental signals into other organs or tissues (see review by Begara-Morales, 2018). Therefore, its absence or limitation may disturb reactions to stresses particularly those linked to protein *S*-nitrosylation. The intracellular level of GSNO is controlled by GSNO formation and its catabolism by GSNOR, which in turn is regulated by PTMs, e.g., *S*-nitrosylation (Guerra et al., 2016) or oxidation (Kovacs et al., 2016). Kubienová et al. (2014) demonstrated stimulation of GSNOR activity in plants exposed to various stressors (low, high temperature, mechanical injury, or pathogens), while Chaki et al. (2011) noticed downregulation of GSNOR after mechanical wounding. Exposition of tomato to *meta*-tyrosine (NPAA released into the environment as root exudates of fescues) led to an increased GSNOR activity and higher abundance of the protein (Krasuska et al., 2017). Although the morphological consequences of CAN supplementation in tomato seedlings were similar to that observed after *meta*-tyrosine (Andrzejczak et al., 2018), its impact on GSNOR was opposite. We noticed slightly higher content of the protein, only small inhibition of the enzyme after short-term treatment with CAN and constant activity as the experiment was prolonged. In addition, expression of *GSNOR* was not affected by CAN. Therefore, we suspect that GSNO level in CAN-treated roots depends rather on its formation than catabolism (**Figure 7**). These results are in agreement with the model proposed by Guerra et al. (2016), suggesting that at low NO concentration in the cells, GSNOR activity is maintained at constitutive level.

### Canavanine Induced Alterations in the Cellular Antioxidant System

The key role of ROS and RNS in plant response to stresses was demonstrated in many experiments and reviewed in details (Molassiotis and Fotopoulos, 2011; Farnese et al., 2016). Their cross-talk has become a more and more fascinating topic, as development in research methodology allows for identification of proteins that are post-translationally modified by ROS or/and RNS. Stress induced by application of allelochemicals is commonly associated with induction of oxidative stress (Gniazdowska et al., 2015). CAN (naturally synthesized in legumes and stored mostly in seeds) is a toxic molecule of well-established negative effect against herbivores (Rosenthal, 2001; Staszek et al., 2017), but its action in plants is only fragmentarily investigated. CAN supplementation resulted in overproduction of H_2_O_2_ and O_2_^•-^ in tomato roots (Krasuska et al., 2016a; Krasuska et al., 2016b), and it was accompanied by elevated activity of ROS-producing enzymes (polyamine oxidase, NADPH oxidase) (Krasuska et al., 2016a). In the current work, we proved that CAN modified cellular antioxidant activity and enzymatic antioxidant system. Relatively small oxidative damages [electrolyte leakage, malondialdehyde (MDA) content, and DNA fragmentation] (Krasuska et al., 2016b) observed in CAN-stressed roots could be a result of the high total antioxidant capacity of the tissue. The elevated total antioxidant capacity in CAN-supplemented seedlings was related to the total thiols concentration (**Figure 7**). Increase of the total thiols was a rapid reaction to CAN, and its gradual increase was characteristic for prolongation of an NPAA treatment. We suspect that CAN leads to increased GSH content. A similar situation was detected in stress induced by some allelochemicals, e.g., a strong influence of *Achillea santolina* L. shoot extract on wheat (*Triticum aestivum* L.) plants was accompanied by accumulation of GSH (Hatata and El-Darier, 2009). It cannot be excluded that relatively high total antioxidant capacity in CAN-exposed roots may be due to a stimulation of biosynthesis of other nonenzymatic antioxidants, particularly phenolic compounds. Phenols accumulation was observed in tomato roots treated with *meta*-tyrosine, and we proposed that it could be regarded as a protective mechanism against stress induced by this NPAA (Andrzejczak et al., 2018).

Plants contain several types of enzymes that are able to modulate ROS level. Among them, SOD is responsible for O_2_^•-^ disproportionation into H_2_O_2_. An activity of this enzyme was lower after CAN treatment. A decline in activity of all tested isoforms, MnSOD, FeSOD, and CuZnSOD, was noticed particularly after 24 h of exposition to CAN. This finding corresponds to the high level of O_2_^•-^ in CAN-supplemented tomato roots (Krasuska et al., 2016a). What more, prolongation of the culture period resulted in a downregulation of most genes encoding SOD, particularly *FeSOD*, *CuSOD*, and *SODCP-2*. *MnSOD* was upregulated by 50-µM CAN both after short and prolonged treatment. As *MnSOD* is highly induced by O_2_^•-^ (Wang et al., 2016), elevated level could be one of the explanations of modification of the MnSOD activity. FeSOD and CuZnSOD are inhibited by H_2_O_2_ (Wang et al., 2016); therefore, accumulation of H_2_O_2_ after CAN application may result in lowering activity of these SOD isoforms. Different isoforms of SOD undergo inactivation by nitration (Holzmeister et al., 2015). Therefore, as CAN induced a transient increase in ONOO^-^ (Krasuska et al., 2016a), the decrease in total SOD activity could be explained by nitration of the enzyme, although we did not identify SOD as a CAN-induced target of this PTMs. A more interesting suggestion for an explanation of lowering total SOD activity by CAN may be the impact of this NPAA on NO synthesis. It was shown that exogenous spermidine (Spd) increased activity of SOD in bluegrass (*Poa pratensis* L.) (Puyang et al., 2015). Spd enhanced NO emission in germinating apple embryos (Krasuska et al., 2014); therefore, we cannot exclude that limitation of NO production by CAN may result in a restriction of SOD activity.

CAT is regarded as the first cellular weapon against H_2_O_2_. Its activity can be decreased by NO donors, e.g., SIN-1 (producing ONOO^-^) (Chaki et al., 2015). In tomato plants, an activity of CAT decreased only after the short period of CAN supplementation, while downregulated expression of *CAT2* and *CAT3* was noticed after both 24- and 72-h CAN application. *CAT1* expression level in control roots was lower than two other genes encoding CATs (data not shown). Thus, we can suspect that although we have observed upregulation of *CAT1* by CAN, it could not influence CAT total activity (sum of activity of CAT1 and CAT2 isoforms). It needs to be mentioned that in roots of Arabidopsis, expression of only *CAT2* (*CAT3* in tomato) and *CAT3* (*CAT2* in tomato) is detected (Mhamdi et al., 2012). Therefore, it is possible that products of these genes (*CAT2* and *CAT3*) are of more importance for regulation of H_2_O_2_ concentration. As a result, downregulation of these genes after CAN application could explain a decrease in CAT activity in tomato roots and H_2_O_2_ accumulation (Krasuska et al., 2016a). In addition, lower than in the control activity of CAT after 24 h of CAN application may be due to putative protein nitration, although as for SOD, we did not find CAT in the group of proteins preferentially nitrated in response to CAN.

GR is one of the enzymes of the enzymatic antioxidant system that sustains the reduced status of GSH and plays a crucial role in maintenance of sulfhydryl (–SH) group. GR has been identified as both nitration and *S*-nitrosylation target (Begara-Morales et al., 2015), although in pea plants in contrast to humans, no effect of these PTMs on activity of the enzyme was shown (Begara-Morales et al., 2016). CAN, in general, had no influence on activity of GR in tomato roots except the drastic drop observed after 24 h of supplementation with CAN at a lower concentration. This phenomenon is hard to explain since downregulation of the genes coding GR was more pronounced after longer CAN treatment. The studies of GR in various plant species have shown an increased GR activity under stresses (Yousuf et al., 2012; Gill et al., 2013). Based on experiments performed using transgenic plants, it is suggested that GR plays an important role in plant resistance to oxidative stress induced by abiotic stress. In addition, it was proved that activity of GR2 is necessary for root growth and maintenance of root apical meristem (Yu et al., 2013). Arabidopsis mutants *miao* (displaying reduction of GR) were characterized by an inhibition of root growth and severe defects in root apical meristem similar to those that were observed in tomato seedlings exposed to CAN for a longer period.

In plants, GPxs are regarded not only as ROS scavenging agents but also as redox sensors taking part in redox transduction signaling pathways as regulators of other regulatory proteins, e.g., transcription factors (Bela et al., 2015; Passaia and Margis-Pinheiro, 2015). GPxs in plants are suggested to be more efficient in reducing peroxides different from H_2_O_2_, e.g., organic hydroperoxides and lipid peroxides. In tomato, activity of GPx was not affected by CAN even after longer period of treatment with NPAA at a high concentration. It may be due to a relatively low abundance of putative substrates, e.g., lipid peroxides, since oxidative damages of membranes were not observed in CAN-supplemented seedlings (Krasuska et al., 2016b). Generally, GPx gene expression levels were higher after 24-h CAN application (with the exception of *GSHPx1*) and declined as the experiment was prolonged. This is in agreement with some observation indicating that GPx mRNA levels usually increase under various biotic and abiotic stresses (as cited by Bela et al., 2015), but it is not the only possible pattern. Arabidopsis mutants *Atgpx1*, A*tgpx4*, *Atgpx6*, *Atgpx7*, and *Atgpx8* had a significantly greater lateral root density than the wild type, similarly as observed in CAN-stressed tomato roots (Krasuska et al., 2016b), suggesting the importance of GPx activity for root architecture (Passaia et al., 2014). What more, transgenic tomato with GPx genes overexpression exhibited a high tolerance to abiotic stress but lower to biotic stresses (pathogens and parasites), probably because of GPx interference with elements of H_2_O_2_-mediated signal transduction under pathogen infection (Herbette et al., 2011). We suspect that the decrease in NO and transient increase in ONOO^-^ formation, as the reaction to CAN application, may impact GPx genes expression in tomato in an atypical way.

### Elements of the Cellular Antioxidant System and Seed Storage Proteins Were Identified as Targets of Tyrosine Nitration in Seedlings Supplemented With Canavanine

In tomato roots, growing in CAN content of 3-NT increased during the culture period. A pattern of nitrated proteins was similar in both CAN-stressed and control plants (Krasuska et al., 2016a). Additional performing immunoprecipitation and Western blot analysis allowed us to distinguish several bands, characterized by stronger reaction with antibodies against 3-NT. Similarly, as in other plant material (Begara-Morales et al., 2016; Kolbert et al., 2017), monodehydroascorbate reductase (MDAR) was identified as nitrated protein in extracts from roots of plant exposed to CAN (10 and 50 µM) for 72 h (**Figure 7**). In tomato roots, MDAR activity was significantly reduced by CAN after 24 and 72 h in concentration-dependent manner (**Table S3**). This observation corresponds well to data on MDAR activity regulation by NO-PTMs. In pea (*Pisum sativum* L.) plants, peroxisomal MDAR was shown to be deactivated by both *S*-nitrolysation and nitration (Begara-Morales et al., 2015). Reduced MDAR activity in CAN-supplemented tomato roots could limit regeneration of ascorbate and lead to disturbance in the glutathione-ascorbate cycle. Among nitrated proteins identified in extracts from roots of CAN-treated plants after 72 h of culture peroxidase 3-like, polyphenol oxidase D were found, which are involved in plant reaction to biotic stresses (herbivores) or have been implicated in the biosynthesis of pigments and other secondary metabolites (Araji et al., 2014). A predicted inhibition of these enzymes being the result of 3-NT formation may induce alterations in secondary metabolites in tomato, mainly in phenylpropanoid pathways. In younger plants after 24 h of CAN supplementation, predominantly seed storage proteins were identified as nitration targets. They are probably preferentially degraded, as is suggested also for carbonylated storage proteins (Job, 2005). Similarly, in apple embryos, legumin A-like protein was nitrated during dormancy alleviation (Krasuska et al., 2016a). A nitration of binding proteins (BiP) localized in the lumen of endoplasmatic reticulum (ER) detected in roots stressed with CAN for short-term may suggest a disruption in secretion of proteins from ER. It is possible that nitrated BiP cannot stabilize proteins in ER or do not prevent aggregation of malformed proteins. The proper function of BiP proteins is necessary for plant immunity or osmotic stress (Carvalho et al., 2014). In addition, it requires ATP, while nitration of phosphoglycerate kinase identified as differentially nitrated protein in CAN-exposed roots could lead to ATP limitation. An interesting observation is CAN induced nitration of mitochondrial prohibitin-1 or prohibitin-3. Although loss of the prohibitin complex in yeast did not affect the mitochondrial membrane potential and respiration, overexpression of prohibitin-1 in endothelial cells decreased the accumulation of ROS, suggesting that prohibitins protect against oxidative stress (Merkwirth and Langer, 2009). Therefore, prohibitin loss of function due to formation of 3-NT may accelerate ROS accumulation in CAN-stressed tomato roots.

### Conclusions

In the current work (**Figure 7**), the inhibition of Arg-dependent NOS-like activity was demonstrated as a direct mode of action of CAN in tomato roots. Limitation in NO synthesis resulted in alteration in cellular antioxidant system; CAN increased antioxidant capacity and the level of sulphydryl groups. Enzymatic antioxidants were suppressed particularly after prolonged culture of seedlings with tested NPAA. Plant supplementation with CAN lowered GSNO accumulation in root tips and does not influenced GSNOR. In seedlings exposed to CAN, we have also shown a new data regarding differentially nitrated proteins. Among them, seed storage proteins (after short-term CAN treatment) and components of the cellular redox system (after prolonged CAN supplementation) were identified. We identified MDAR as a protein nitration target and demonstrated that the activity of the enzyme in roots was lowered after CAN application into the culture medium (**Figure 7**).

## Data Availability

The raw data supporting the conclusions of this manuscript will be made available by the authors, without undue reservation, to any qualified researcher.

## Author Contributions

Conceptualization, AG and PS; Methodology, PS and UK; Investigation, PS; Data Curation, PS and UK; Writing—Original Draft Preparation, AG and PS; Writing—Review and Editing, AG and PS; Supervision of the experimental work, UK, JF, and KO-K; Project Administration, AG and PS; Funding Acquisition, AG and PS. All authors read and approved the final manuscript.

## Funding

The work was done during realization of the project financed by National Science Centre Poland 2014/13/B/NZ9/02074 given to AG and the project for young scientist financed by WULS-SGGW 505-10-010200-Q00212-99 given to PS. The funders had no role in the study design, data collection and analysis, or preparation of the manuscript.

## Conflict of Interest Statement

The authors declare that the research was conducted in the absence of any commercial or financial relationships that could be construed as a potential conflict of interest.

## Abbreviations

CAT, catalase; DPPH, 2,2-diphenyl-1-picrylhydrazyl; GPx, glutathione peroxidase; GR, glutathione reductase; GSH, glutathione reduced form; MDAR, monodehydroascorbate reductase; PTM, post-translational modification of protein; NPAA, Nonproteinogenic Amino Acid; O_2_^•-^, superoxide radical; ROS, Reactive Oxygen Species; SOD, superoxide dismutase.
