# Supplementary material for: Canavanine-Induced Decrease in Nitric Oxide Synthesis Alters Activity of Antioxidant System but Does Not Impact S-Nitrosoglutathione Catabolism in Tomato Roots
Source: Front Plant Sci. 2019 Sep 20;10:1077. doi: 10.3389/fpls.2019.01077 (PMC6763595; doi:10.3389/fpls.2019.01077)
Supplement: Supplementary file 1 [file DataSheet_1.pdf]

Table S1. List of the primers used for RT-qPCR experiments

Gene name	Gene ID (NCBI)	Primers sequences 5'-3'	PCR product length (bp)
CAT1	543990	F: TCCCAGTTAATGCTCCCAAG<br>R: ACCTCGAGGGCAAATAATCC	102
CAT2	543585	F: ATCCTCGTGGTTTTGCTGTC<br>R: TGAAGAAGACGGGGAAGTTG	81
CAT3	101259333	F: ACAAAGCTGGGAAAGCACAG<br>R: TCCTTGGTGGCATGACTATG	121
MNSOD	101256386	F: CAGAAGGTGCTGCTTTACAGG<br>R: AAGGGTCCTGATTTGCAGTG	99
CUSOD2	101264296	F: GCAATCCAATGGTGTTACCC<br>R: ATGGAAAGCGTGAAGACCTG	70
FESOD	544259	F: TAACAATGGTGCTCCCCTTC<br>R: CGTTGGGCTTCATAGATTCC	86
SODCP.2	543981	F: AATGTTGAGGGGGTTGTCAC<br>R: ATGAAACCCGTGAAGTCCAG	99
SOD3	101256231	F: ACACCTGCTAAGCCAAAACC<br>R: CCAATGGCTGCTACAAATCC	116
GPXle-1	544197	F: AAATGGTCTGCACTCGCTTC<br>R: CTCCAAAGAACCCACCTTTG	120
GPXle-2	101267098	F: GGATGCAAAGGGAAATGATG<br>R: GACGCAACATTCACGATCAG	72
GPx	544261	F: GATGTGAACGGGGATAATGC<br>R: ACAGCACTTCCAAGGAAACC	77
pGPx8	101267388	F: CGCGACAGGAAATGATGTAG<br>R: GTTAGTCATCCCGCATTTGG	88
GSHPx1	101267727	F: ACTATCACTGCCCAAACCTCTC<br>R: TAAAGCAACCGAGAGAGAGACC	94
GSHPx2	101248896	F: TGACAAGGTCGACGTGAATG<br>R: CACCTAAAAATCCTCCTGCAC	80
GR	100301931	F: CTCCTCGGCATTACGATTTC<br>R: CTCACAAACAGCAACCGAAG	107
GR1	100301935	F: CGAGCTAGTCGGTTTTCAGC<br>R: CCCATAGGCTGCTCCATAAA	159
GSNOR	100750249	F: CTGGAGTGGGAGTTATGATGAA<br>R: CCTCCGCCACAGCAAGACCAACT	279
EF1α	101244084	F: GGTCATCATCATGAACCATCC<br>R: CATACCAGCATCACCGTTCTT	175
PP2Acs	001247587.2	F: CGATGTGTGATCTCCTATGGTC<br>R: AAGCTGATGGGCTCTAGAAATC	149

Table S2. Details of MALDI MS/MS identification after trypsin in-gel digestion of the nitrated proteins of tomato roots treated with CAN (10, 50 µM) for 24 or 72 h. Scores and threshold for significant homology or identity by MASCOT MS/MS Ions Search (Matrix-Science)

	**Culture period (h)**	**Protein band (Fig. S4)**	**Description**	**NCBI ID**	**Number of identified peptides**	**Score of identified peptides (in parenthesis)**	**Significant homology or identity**
**CAN 10 µM**	24	a	Luminal-binding protein 5	XP_004234985.1	3	1 (39)<br>2 (64)<br>3 (64)	>36<br>>56<br>>34
		b	Phosphoglycerate kinase, chloroplastic	XP_004243968.1	2	1 (33)<br>2 (59)	>29<br>≥57
		c	11s globulin seed storage protein 2-like (fragment)	XP_004247523.1	3	1 (66)<br>2 (56)<br>3 (49)	≥57<br>>46<br>>39
		d	12s seed storage protein CRA1-like (fragment)	XP_004246943.1	2	1 (61)<br>2 (39)	>37<br>>37
	72	e	Polyphenol oxidase D, chloroplastic	NP_001334885.1	4	1 (31)<br>2 (31)<br>3 (49)<br>4 (57)	>21<br>>25<br>>21<br>>20
		f	Prohibitin-3 mitochondrial	XP_004250114.1	3	1 (62)<br>2 (35)<br>3 (41)	>56<br>>21<br>>36
		g	Monodehydroascorbate reductase	NP_001318117.1	4	1 (38)<br>2 (69)<br>3 (43)<br>4 (36)	>45<br>≥57<br>>39<br>>35
		h	Phospoglycerate kinase, chloroplastic	XP_004243968.1	2	1 (62)<br>2 (47)	>33<br>>46
**CAN 50 µM**	24	i	Aconitate hydratase, cytoplasmic	XP_004251517.2	3	1 (75)<br>2 (34)<br>3 (52)	≥70<br>>23<br>>41
		j	Luminal-binding protein 5	XP_004234985.1	2	1 (39)<br>2 (64)	>36<br>≥56
		k	Phosphoglycerate kinase. chloroplastic	XP_004243968.1	2	1 (33)	>29

						2 (59)	>57
		l	Vivilin precursor (fragment)	NP_001308118.1	2	1 (54)<br>2 (82)	>47<br>>70
		m	Prohibitin-1, mitochondrial-like	XP_004251498.1	4	1 (87)<br>2 (43)<br>3 (57)<br>4 (26)	>69<br>>39<br>>34<br>>38
		n	11s globulin seed storage protein 2-like<br>(fragment)	XP_004247523.1	3	1 (57)<br>2 (55)<br>3 (41)	>36<br>>46<br>>39
		o	12s seed storage protein CRA1-like<br>(fragment)	XP_004246943.1	2	1 (61)<br>2 (39)	>37<br>>37
	72	p	Peroxidase 3-like	XP_006367274.1	2	1 (57)<br>2 (23)	>55<br>>18
		r	Polyphenol oxidase D, chloroplastic	NP_001334885.18	3	1 (23)<br>2 (41)<br>3 (48)	>21<br>>20<br>>46
		s	Monodehydroascorbate reductase	NP_001318117.1	4	1 (40)<br>2 (44)<br>3 (69)<br>4 (40)	>45<br>>33<br>>57<br>>45
		t	Phospoglycerate kinase, chloroplastic	XP_004243968.1	3	1 (59)<br>2 (21)<br>3 (64)	>57<br>>20<br>>53

Monodehydroascorbate reductase (MDAR) activity

Method - determination of MDAR activity in roots extracts

Activity of MDAR was measured according to Hossain et al. (1984) with some modifications by Leterrier (2005). Root were homogenized in an ice bath with 50 mM Tris-HCl pH 7.8, 10% (w/v) glycerol, 5 mM DTT, 0.2% Triton X-100 mM, 1% (v/v) protease inhibitor cocktail (Sigma-Aldrich), 5% (w/v) PVPP and centrifuged at 10,000 *g* 15 min. The collected supernatant was concentrated by PES, 3K MWCO (Thermo ScientificTM) at 10,000 *g* for 30 min. An assay mixture (250 µl) contained: 25 µl of protein extract, 0.2 mM NADH and 1 mM NADH in 50 mM Tris-HCl pH 7.6. The reaction was started by an addition of 0.2 U of ascorbate oxidase (A0157 Sigma-Aldrich). MDAR activity was measured as absorbance decrease at 340 nm for 3 min at 25°C, using a microplate reader (Sunrise, Tecan). An activity was calculated using the extinction coefficient $\varepsilon = 6.22\ mM^{-1}\ cm^{-1}$ and expressed as nmol $min^{-1}mg^{-1}$protein.

Result

Activity of MDAR in control tomato roots growing in water increased as the culture period was prolonged. In roots of CAN supplemented seedlings MDAR activity was lower than in control. After 24 h MDAR activity was inhibited in 20% by 50 µM CAN, whereas after 72 h inhibition was 30% and 50 % after 10 and 50 µM CAN application respectively.

Table S3. Activity of MDAR in extracts from roots of the control seedlings grown in water and roots of seedlings treated with CAN (10, 50 µM) for 24 and 72 h. Values are average ±SD of 3-4 repetitions. Asterisks (*) indicate significance between treatments and the control at the same time of culture period at $P \leq 0.05$, based on Student's test

Plant treatment	MDAR activity (nmol NADH min^{-1} mg^{-1} protein)	
	24 h	72 h
Control (water)	0.061 ± 0.004	0.074 ± 0.005
CAN 10 µM	0.056 ± 0.003	0.052 ± 0.003*
CAN 50 µM	0.048 ± 0.003*	0.037 ± 0.004*

Hossain, M. A., Nakano, Y., and Asada, K. (1984). Monodehydroascorbate reductase in spinach chloroplasts and its participation in regeneration of ascorbate for scavenging hydrogen peroxide. *Plant Cell Physiol.* 25, 385–395. doi:10.1093/oxfordjournals.pcp.a076726.

Leterrier, M. (2005). Peroxisomal monodehydroascorbate reductase. Genomic clone characterization and functional analysis under environmental stress conditions. *Plant Physiol.* 138, 2111–2123. doi:10.1104/pp.105.066225.

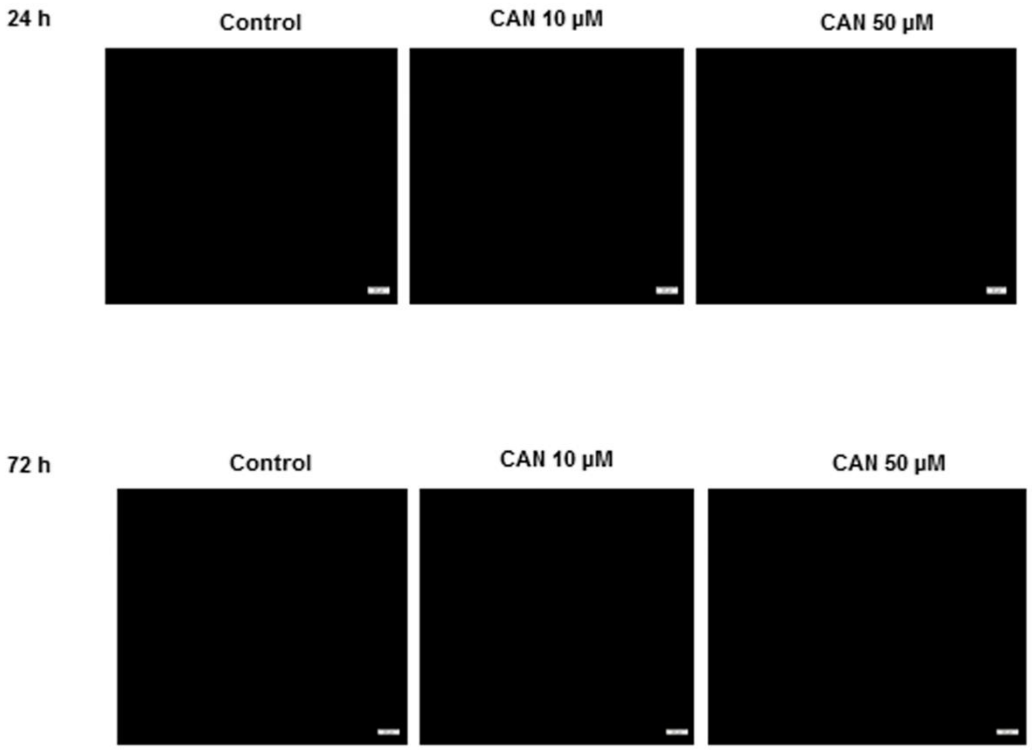

Fig. S1. Visualization of GSNO-related red fluorescence signal in root apex of tomato seedlings. Controls for background staining performed by replacing the primary antibody with the incubation buffer. Scale bars 20 μm.

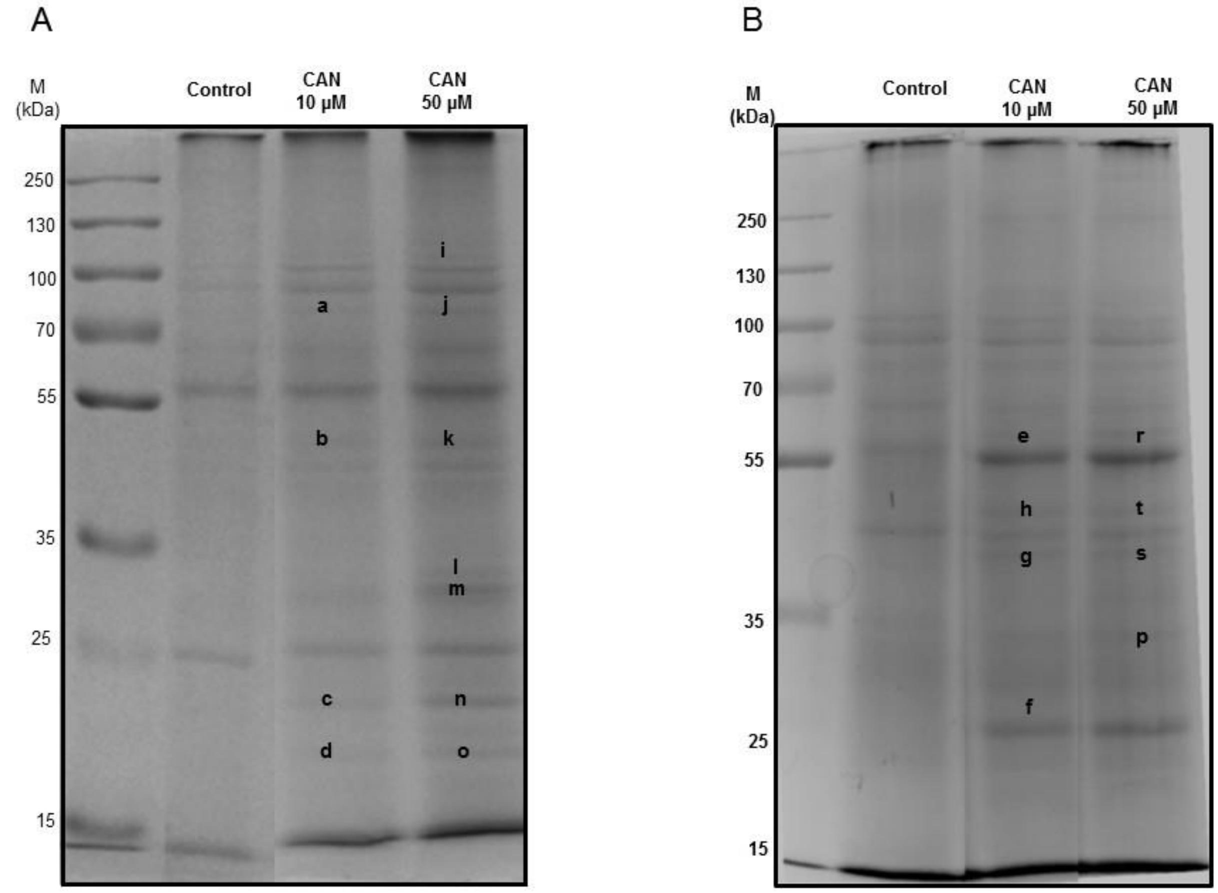

Fig. S2. Pattern of 3-NT modified proteins (after immunoprecipitation) in extract of tomato roots after 24 h (A) or 72 h (B) of the culture. Control - plants growing in water, CAN 10 µM - seedlings treated with 10 µM CAN, CAN 50 µM - seedlings treated with 50 µM CAN. Molecular standard (M) in kDa are indicated on the left side. Gels were stained with Coomassie. Protein bands indicated by small letters (a-t) (Table S2) were cut out and identified by MALDI MS/MS (Table 3).

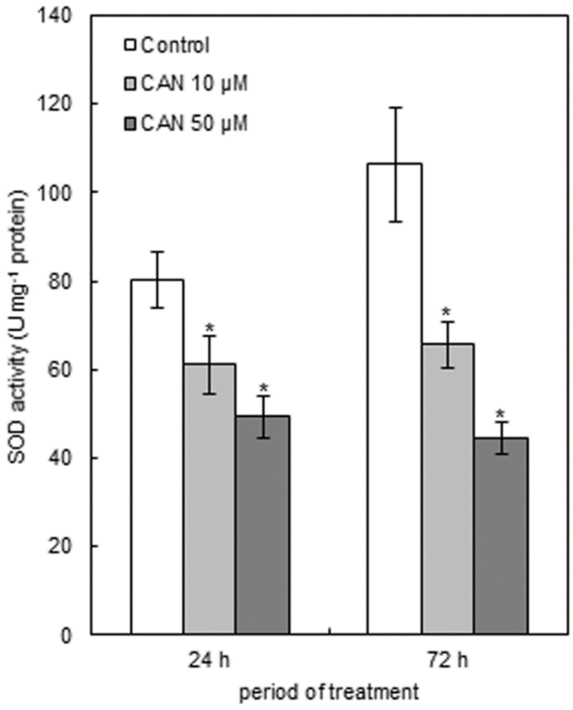

Fig. S3. Activity of SOD in extracts from roots of the control seedlings growing in water and roots of seedlings treated with CAN (10, 50 µM) for 24 and 72 h. Values are average ± SD of at least 3 independent experiments and 3 biological repetitions each. Asterisks (*) indicate significance between treatments and the control at the same time of culture period at $P \leq 0.05$, based on Student's test.

Supplementary material

A

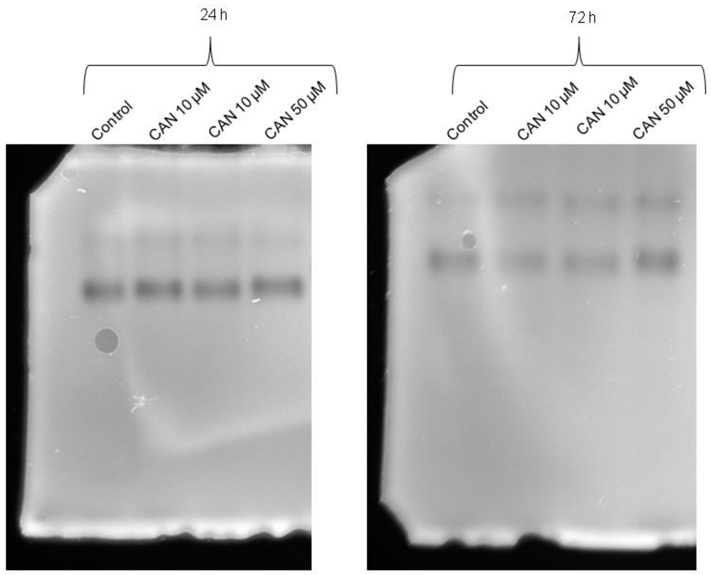

B

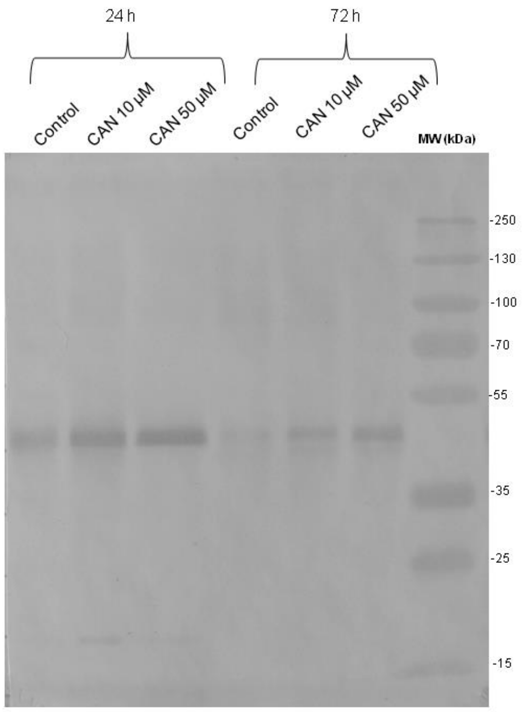

Original images for the gel Fig.5B in the manuscript (A) and the blot Fig.5C in the manuscript (B).

PageRuler™ Plus Prestained Protein Ladder (Thermo Scientific™, 26620) was used (B).
